# Heat Stress Promotes Fibroblast‐Derived WNT5A Secretion Through m^6^A Modification to Activate Melanogenesis

**DOI:** 10.1002/advs.76820

**Published:** 2026-07-27

**Authors:** Yuanyuan Wan, Yaqing Wen, Yushan Zhang, Lan Zhang, Jianjian Zhu, Zhen Tang, Ling Jiang, Chuhan Fu, Shu Zhou, Keyi Zhang, Jiangfeng Huang, Li Lei, Jing Chen, Qinghai Zeng

**Affiliations:** ^1^ Department of Dermatology, Third Xiangya Hospital Central South University Changsha Hunan People's Republic of China; ^2^ Medical Education Center of Jinan University Guangzhou People's Republic of China; ^3^ Department of Dermatology, Xiangya School of Medicine, Changde Hospital Central South University (The First People's Hospital of Changde City) Changde People's Republic of China

**Keywords:** fzd10, heat stress, melanogenesis, n6‐methyladenosine, paracrine function, wnt5a

## Abstract

As the impact of global warming continues to intensify, the effects of heat stress on the skin are becoming increasingly evident. Growing clinical evidence suggests that heat stress can induce cutaneous hyperpigmentation. However, the underlying mechanisms remain incompletely understood. In this study, we demonstrate that heat stress promotes repigmentation in vitiligo lesions and induces melanogenesis in depigmented mouse skin. Mechanistically, we reveal that heat stress upregulates WNT5A expression and paracrine secretion in dermal fibroblasts (FB) through METTL3‐mediated N^6^‐methyladenosine (m^6^A) modification. The stability of WNT5A mRNA is regulated through a YTHDC1‐dependent pathway. Treatment with recombinant WNT5A protein significantly increases melanin content in melanocytes (MC), MNT1 cells, and human skin explants. Furthermore, WNT5A induces the expression of both β‐catenin and its receptor FZD10 in MC. Functional rescue experiments confirm that FZD10 knockdown abolishes WNT5A‐ and heat stress‐induced β‐catenin activation, thereby attenuating melanogenesis. Collectively, our findings elucidate a novel mechanism whereby heat stress triggers fibroblast‐derived WNT5A secretion via METTL3/YTHDC1‐dependent m^6^A methylation to promote melanogenesis. This study provides novel insights into the pathogenesis of heat‐induced skin pigmentation disorders, and reveals the potential of thermotherapy as a promising avenue for treating hypopigmentary diseases.

## Graphical abstract

Heat stress promotes WNT5A secretion from fibroblasts via METTL3/YTHDC1‐dependent m^6^A methylation. Subsequently, WNT5A binds to FZD10 on melanocytes, activating β‐catenin‐driven melanogenesis.

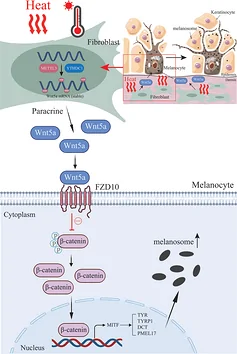

## 1 Introduction

As the largest organ directly exposed to the external environment, the skin is highly susceptible to environmental factors such as ultraviolet (UV) radiation, heat, and CO_2_ [1]. With the escalating impact of global warming, the effects of heat stress on the skin have become increasingly evident [2]. Accumulating evidence suggests that heat stress can induce cutaneous melanogenesis. Chronic and repeated exposure to subthreshold‐intensity heat that is not hot enough to cause a burn may lead to persistent reticular hyperpigmentation, clinically known as erythema ab igne [3, 4]. Additionally, melasma, a common acquired hyperpigmentation disorder, has been closely associated with heat stress [5]. Notably, fire needle therapy has shown promising clinical efficacy with in treating vitiligo [6]. From the perspective of melanocytes themselves, our team previously demonstrated that heat stress upregulates CX3CL1 via the MYC‐WDR5‐H3K4me3 axis, thereby activating the CX3CL1/CX3CR1‐JNK signaling pathway to promote melanogenesis [7]. Furthermore, we also demonstrated that heat stress activates the TRPV3/Ca^2^^+^/Hedgehog signaling pathway, enhancing paracrine signaling in keratinocytes and subsequently promoting melanogenesis [8]. However, research on the mechanisms underlying heat stress‐induced skin pigmentation remains limited, and the precise regulatory pathways are not fully elucidated. Investigating these mechanisms may provide novel therapeutic targets for pigmentary disorders.

Skin fibroblasts (FB), the principal cells in the dermis, play a dynamic role in epidermal melanogenesis. They contribute to extracellular matrix (ECM) production and secrete various melanogenic modulators, including stem cell factor (SCF), basic fibroblast growth factor (bFGF), and hepatocyte growth factor (HGF) [9]. Recent studies have shown that FB‐derived secreted frizzled‐related protein 2 (sFRP2) regulates the Wnt/β‐catenin pathway, upregulating microphthalmia‐associated transcription factor (MITF) and tyrosinase (TYR) to stimulate melanogenesis in human melanocytes [10]. Furthermore, thermal stimulation has been reported to modulate FB cells function via the cation channel TRPV1, suggesting that FB cells exhibit regulatory cascades similar to keratinocytes upon heat exposure [11]. Nevertheless, the role of FB cells in heat stress‐induced melanogenesis remains unclear.

The canonical Wnt/β‐catenin pathway positively regulates melanogenesis [12]. Upon Wnt ligand binding to Frizzled receptors, β‐catenin phosphorylation is inhibited, preventing its degradation and leading to cytoplasmic accumulation and nuclear translocation [13]. In the nucleus, β‐catenin enhances the expression of MITF, promoting melanocyte survival, proliferation, and melanogenesis [12, 13]. WNT5A, a member of the Wnt protein family, exerts its effects through either the canonical Wnt/β‐catenin pathway or non‐canonical Wnt signaling, depending on receptor binding [14]. Although WNT5A has been implicated in melanocyte differentiation and maturation via the canonical Wnt pathway [15], its precise mechanism remains incompletely understood. A transcriptomic analyses of melasma‐affected skin revealed Wnt pathway enrichment, altered expression of melanogenesis‐related genes, and upregulated WNT5A—particularly in the basal layer and perifibroblast regions [16]. These findings suggest a potential role for WNT5A in melanogenesis, yet its involvement in heat stress‐induced pigmentation has not been explored.

N^6^‐methyladenosine (m^6^A), one of the most prevalent epigenetic modification in eukaryotic mRNA, plays a pivotal role in post‐transcriptional regulation by influencing RNA maturation, nuclear export, stability, and translation efficiency [17, 18, 19, 20]. The dynamic regulation of m^6^A is orchestrated by methyltransferases (“writers”), demethylases (“erasers”), and m^6^A‐binding proteins (“readers”). Emerging evidence indicates that heat exposure alters m^6^A modification patterns. For instance, Heat shock (42°C) induces the upregulation of YTHDF2 expression and its translocation from the cytoplasm to the nucleus. It also restricts the demethylation of the 5’UTR of mRNA by FTO, thereby increasing the methylation level of the 5’UTR in heat shock‐induced mRNA and initiating cap‐independent translation [21]. Additionally, YTHDC1 facilitate the co‐transcriptional induction of heat shock proteins in a METTL3‐dependent manner during heat stress, highlighting the critical role of m^6^A signaling in heat‐induced gene reprogramming [22]. Moreover, heat‐triggered m^6^A hypermethylation activates the NLRP3 inflammasome, impairing spermatogenesis in mice [23]. Despite these advances, whether m^6^A modifications contribute to heat stress‐induced melanogenesis remains unknown.

This study investigated the effects and mechanisms of heat stress on skin melanogenesis from the perspective of fibroblast paracrine signaling. Initially, applying thermotherapy to vitiligo patients and subjecting depigmented mouse models to heat stress, we demonstrate that heat stress promotes melanin regeneration. Subsequent spatial transcriptomic analysis of depigmented mouse skin tissue suggests potential roles of WNT5A, its receptor FZD10, and m^6^A modification in heat stress‐induced melanogenesis. Through in vitro experiments, we unveil a novel mechanism wherein heat stress upregulates FB‐derived WNT5A via METTL3/YTHDC1‐mediated m^6^A methylation, which subsequently activates the β‐catenin pathway through FZD10 to drive melanogenesis.

## 2 Results

### 2.1 Heat Stress Can Promote Melanogenesis in Depigmented Skin

We confirmed that heat stress at 41°C could significantly induce skin pigmentation (Figure S1A), which was consistent with our previously published articles [8]. To investigate whether heat stress can promote pigmentation in depigmented skin, six patients diagnosed with vitiligo, based on clinical and reflectance confocal microscopy findings, were included in the study. Clinical characteristics and conventional therapy during follow‐up of patients were summarized in Table S1. In the same patient, the skin lesions were divided into a control group and a thermotherapy group. The control group received conventional therapy, whereas the thermotherapy group received local heat stress treatment (41°C) in addition to conventional treatment. The average area at the baseline in the control group was 3.96 ± 1.15 cm^2^ and decreased to 2.78 ± 0.95 cm^2^ at the follow‐up, revealing an average response of 29.5%. The average area at the baseline in the thermotherapy group was 4.56 ± 0.92 cm^2^ and decreased to 2.23 ± 0.76 cm^2^ at the follow‐up, revealing an average improvement of 54.2% (Figure 1A). And the treatment response of skin lesions in the thermotherapy group was significantly higher than that in the conventional therapy group (Figure 1B). One patient with multiple white spots on the right upper limb achieved rapid repigmentation and got 100% repigmentation in the thermotherapy area (Figure 1C). A repigmentation greater than 50% was observed in the thermotherapy area from three patients. The above clinical observations have demonstrated that heat stress can induce pigmentation in depigmented skin of vitiligo.

**FIGURE 1:**
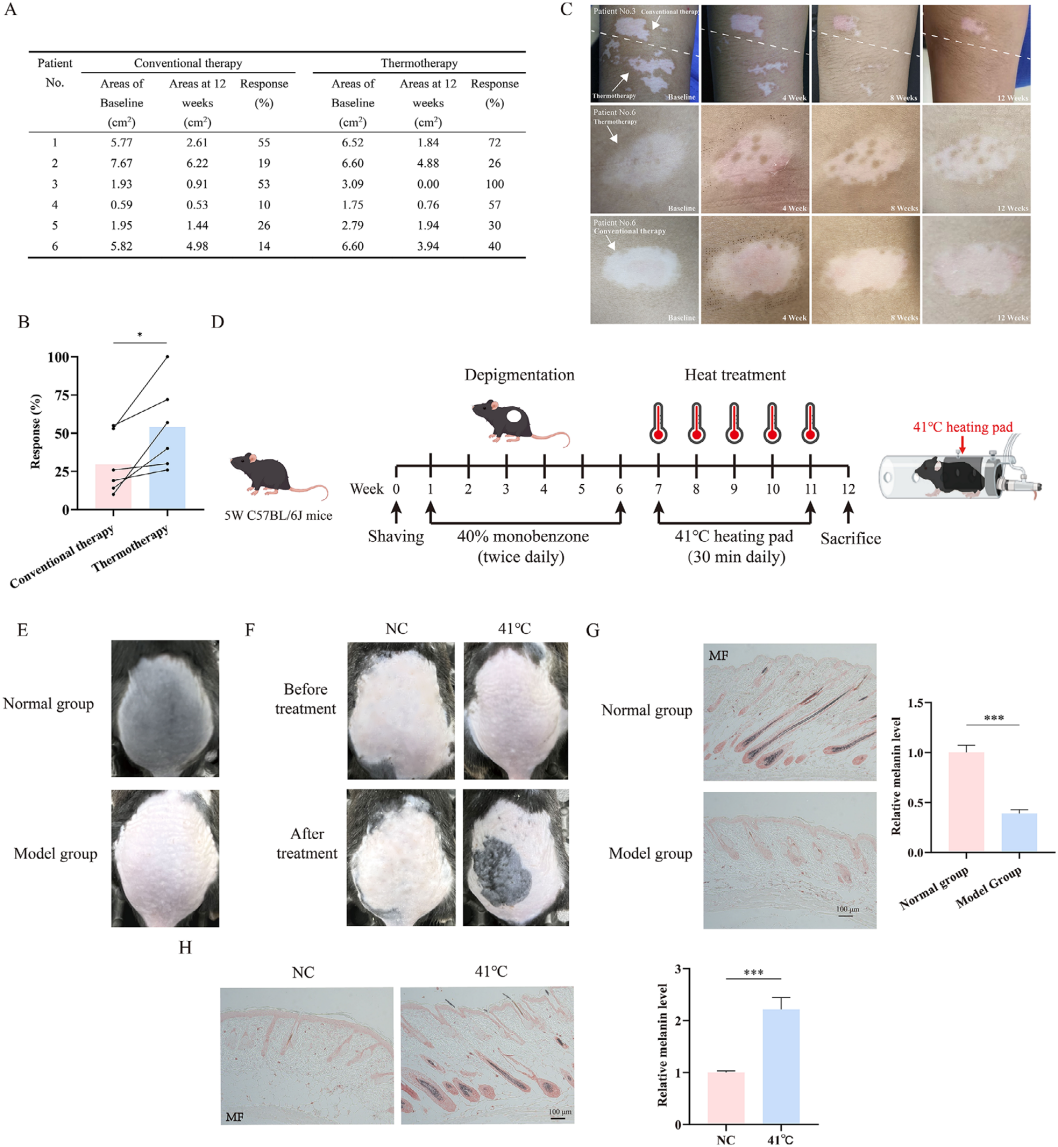
Heat stress can promote melanogenesis in depigmented skin. (A) Repigmentation characteristic of patients with vitiligo. (B) Comparison of response rate between conventional therapy and thermotherapy. Statistical significance was determined by Paired Student's t‐test (*p <0.05). (C) Repigmentation of patients No.3 and No.6 during treatment. (D) Schematic of the establishment of depigmented mice and subsequent heat treatment. (E) Gross morphological changes in the skin of the depigmentation mouse model. (F) Comparison of skin appearance in depigmented mice before and after heat treatment. (G) Representative Masson‐Fontana staining of mouse skin tissue from the normal and model groups (scale bar = 100 µm). Statistical significance was analyzed using Student's t‐test (***p < 0.001). (H) Representative Masson‐Fontana staining of skin tissue from depigmented mice before and after heat treatment (scale bar = 100 µm). Statistical significance was analyzed using Student's t‐test (***p < 0.001).

We subsequently developed a depigmented mouse model to systematically examine heat stress effects on hypopigmented skin. During heat treatment of these mice, we ensured that the skin temperature remained stable at approximately 41°C without inducing systemic stress, as shown in Figure S1B,C. The overall experimental workflow for the animal study is illustrated in Figure 1D. Five‐week‐old C57BL/6J mice were used to establish the depigmentation model. Masson‐Fontana melanin staining was performed to assess the level of depigmentation in the hair follicles, and the results are shown in Figure 1E,G. No difference in the dorsal hair follicle phenotype was observed between female and male mice, and both showed marked hypopigmentation. After heat treatment of the depigmented mice, we found that the melanin content in the hair follicles of the 41°C group was significantly increased compared to the NC group, as shown in Figure 1F,H.

### 2.2 Heat Promotes Expression of Key Melanogenesis Genes

To investigate the potential mechanisms underlying heat stress–induced skin hyperpigmentation, spatial transcriptomic analysis was performed on skin samples from the NC and 41°C groups (Figure 2A). Following quality control (Figure S2A–D), we integrated unsupervised clustering (Figure S2E), t‐SNE/UMAP visualization with batch correction (Figure S2F), and H&E‐guided histological annotation (Figure 2B) to classify the data from both groups into four histological categories: epidermis, dermis, fat, and muscle (Figure S2G). The spatially localized expression revealed that the expression levels of key melanogenesis‐related genes—Tyr, Tyrp1, Pmel, and Dct—were significantly elevated in the 41°C group compared with the NC group (Figure 2C). These findings suggest that heat exposure promotes the upregulation of genes involved in melanogenesis. By screening the differentially expressed genes (DEGs) between the NC group and the 41°C group, followed by GO and KEGG enrichment analyses, we found that the GO database was enriched in terms related to epidermis development, hair cycle, and cell‐cell junction organization, while the KEGG database was enriched in the melanogenesis‐related pathway as well as the Wnt, PI3K‐Akt, MAPK, and Hippo signaling pathways (Figure 2D). Differential expression analysis of genes encoding upstream ligands and receptors involved in melanogenesis‐related pathways (Table S2) revealed a significant upregulation of Wnt signaling components in the 41°C group compared with the NC group (Figure 2E). Based on these findings, we further analyzed the expression changes of WNT family members and their receptors. The results showed that WNT5A and its receptor FZD10 exhibited the most pronounced differences between the two groups (Figure 2E–G and Figure S2H). Moreover, WNT5A and FZD10 showed the strongest correlations with key melanogenesis‐related genes, including Mlana, Tyr, Tyrp1, Dct, Mitf, Pmel, and Myo5a (Figure S2I). These findings suggest that the WNT5A‐FZD10 axis may play an important regulatory role in heat stress‐induced melanogenesis.

**FIGURE 2:**
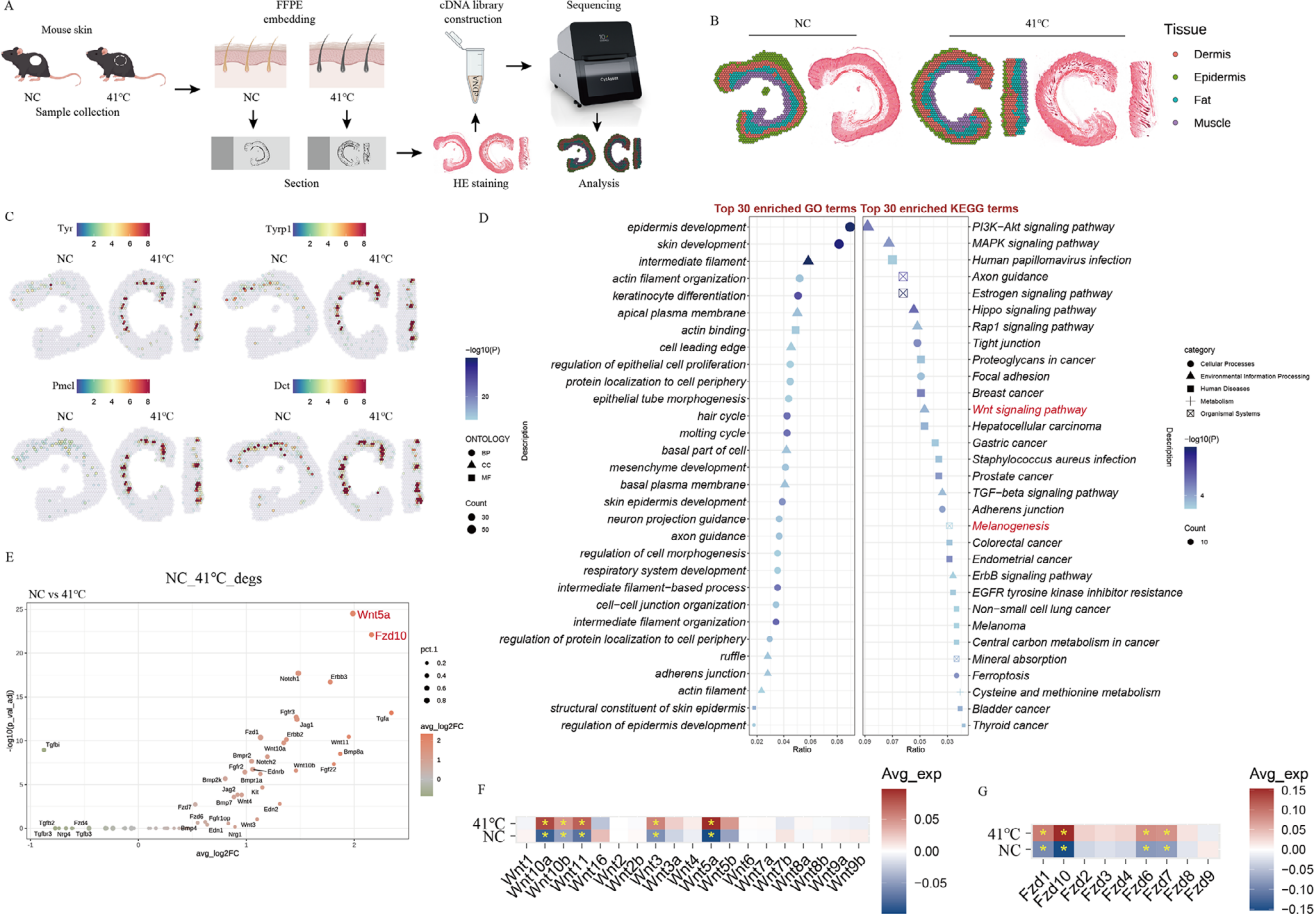
Heat stress promotes elevated expression of melanogenic genes and WNT5A. (A) The procedures of mouse skin sample preparation and RNA sequencing. (B) Spatial distribution of histological types and corresponding H&E staining images. (C) Heat stress promotes up‐regulation of the expression of key melanogenesis genes (Tyr, Tyrp1, Pmel, Dct). (D) Top 30 pathways of GO, KEGG enrichment results. (E) Volcano plot of differentially expressed ligand–receptor pairs related to melanogenesis pathways. (F) Heatmap of Wnt protein expression in the NC and 41°C groups (*p < 0.05). (G) Heatmap of Fzd receptor expression in the NC and 41°C groups (*p < 0.05).

### 2.3 WNT5A Induces Melanogenesis

Through the spatial transcriptomic analysis described above, we found that WNT5A and FZD10 exhibited the strongest correlation with melanogenesis‑related genes (Figure 3A). To further investigate the specific role of WNT5A in melanogenesis, we performed a stratified analysis of the spatial transcriptomic data from the mouse model. This analysis revealed that WNT5A, Fzd10, and other key melanogenesis regulatory factors were enriched in the hair follicle region and exhibited upregulated expression following heat treatment (Figure 3B). Subsequently, we analyzed the expression profiles of these genes in a human melasma dataset (GSE72140) to validate the association between WNT5A and melanogenesis in human skin samples. Differential expression analysis between melasma and normal groups, followed by GO and KEGG enrichment, revealed significant enrichment of the Wnt signaling and melanogenesis pathways (Figure S3A). Expression of WNT5A and melanogenesis‐related regulatory genes (MLANA, TYR, TYRP1, DCT, MITF, PMEL, and MYO5A) was markedly upregulated in melasma samples (Figure S3B,C). Moreover, WNT5A expression showed a positive correlation with the melanogenesis‐related gene set (R = 0.51, *p* = 0.011; Figure S3D). Based on the average expression level of TYR, melasma samples were stratified into TYR‐high and TYR‐low groups. Differential expression and enrichment analyses revealed persistent enrichment of the Wnt signaling pathway and melanogenesis modules between the two groups (Figure S3E), with WNT5A markedly upregulated in the TYR‐high group (Figure S3F). Both spatial and bulk transcriptomic analyses consistently implicated WNT5A is closely associated with melanogenesis.

**FIGURE 3:**
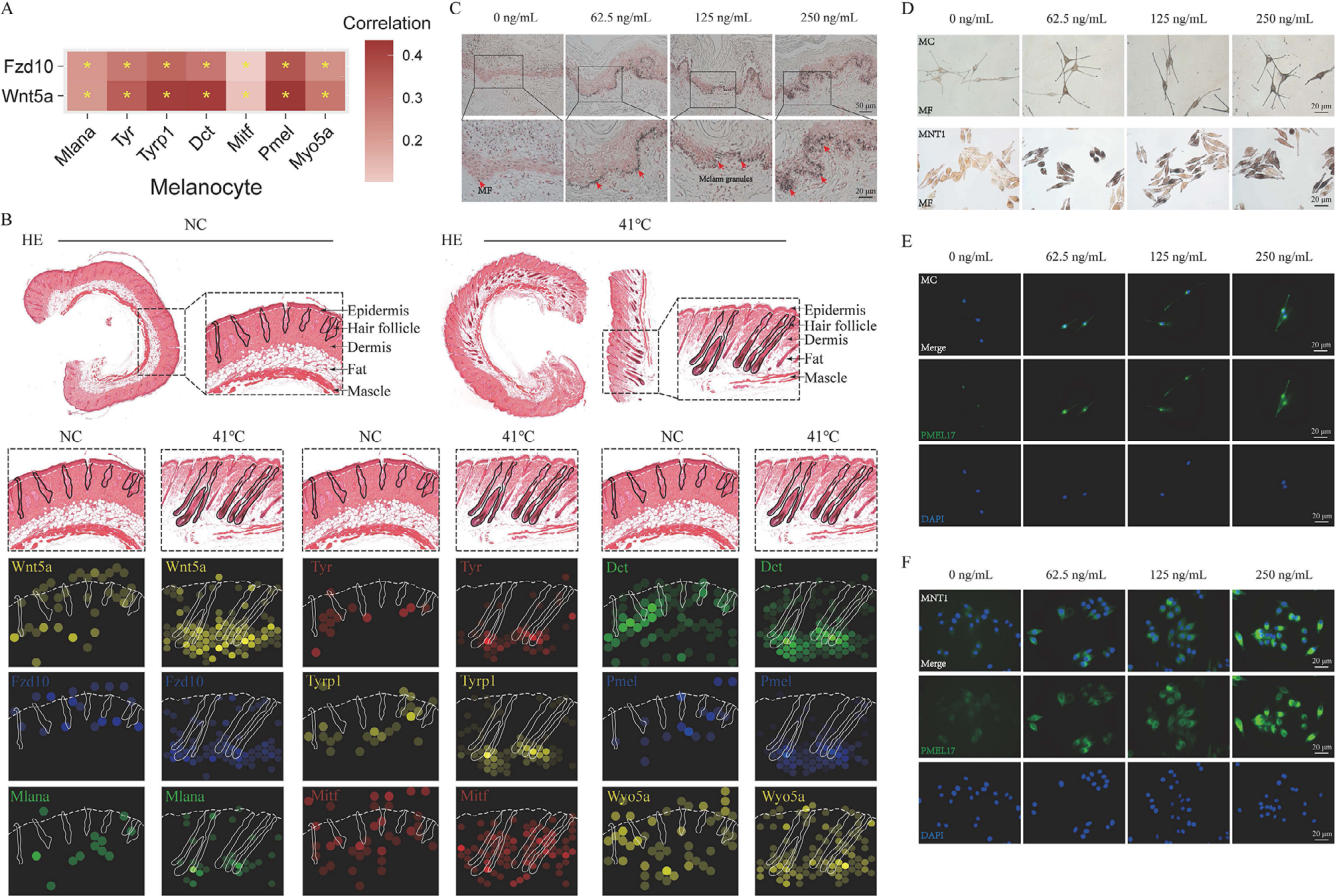
WNT5A correlates with melanogenesis. (A) Correlation between Wnt5a/Fzd10 and melanogenesis‐related regulatory genes (Pearson correlation analysis, *p < 0.05). (B) Colored dot visualization of Wnt5a, Fzd10, and melanogenesis‐related genes (Mlana, Tyr, Tyrp1, Dct, Mitf, Pmel, and Myo5a) in hair follicle regions of NC and 41°C groups. (C) Masson‐Fontana staining showing melanin content in human skin explants after 5 days of WNT5A treatment (scale bar = 50 µm, 20 µm). (D) Masson‐Fontana staining showing melanin content in MC and MNT1 cells after 2 days of WNT5A treatment (scale bar = 20 µm). (E) Immunofluorescence showing PMEL17 expression in MC cells after 2 days of WNT5A treatment (scale bar = 20 µm). (F) Immunofluorescence showing PMEL17 expression in MNT1 cells after 2 days of WNT5A treatment (scale bar = 20 µm).

Subsequently, we further validated the effect of WNT5A on melanogenesis. Human skin tissues, MC, and MNT1 cells were treated with recombinant WNT5A protein at varying concentrations (0, 62.5, 125, and 250 ng mL^−1^) for 5, 2, and 2 consecutive days, respectively. Masson‐Fontana staining revealed that melanin content was significantly increased in WNT5A‐treated skin tissues, MC, and MNT1 cells, compared with the control group (0 ng mL^−1^) (Figure 3C,D). Immunofluorescence staining further demonstrated that exogenous WNT5A enhanced the expression of the melanosome‐specific marker PMEL17 in MC and MNT1 cells (Figure 3E,F). Moreover, in MNT1 cells, TYR probe staining indicated that exogenous WNT5A markedly elevated TYR activity, as evidenced by increased yellow fluorescence (Figure S4A), and upregulated the expression of TYRP1 (Figure S4B).

### 2.4 WNT5A Activates the FZD10/β‐Catenin Pathway to Induce Melanogenesis

Since WNT5A is a component of the Wnt signaling pathway, which was observed to be enriched in both spatial transcriptomic and melasma transcriptomic analyses (Figure 2D and Figure S3A), we sought to determine whether WNT5A promotes melanogenesis via the classical Wnt signaling pathway. MC and MNT1 cells were treated with recombinant WNT5A protein at various concentrations (0, 62.5, 125, and 250 ng mL^−1^) for 2 days. The results demonstrated that WNT5A promoted the nuclear expression of β‐catenin in MC cells (Figure 4A). Furthermore, in MNT1 cells, WNT5A significantly enhanced β‐catenin‐dependent TCF/LEF transcriptional activity (Figure 4B) as well as markedly increasing total β‐catenin protein levels (Figure 4C).

**FIGURE 4:**
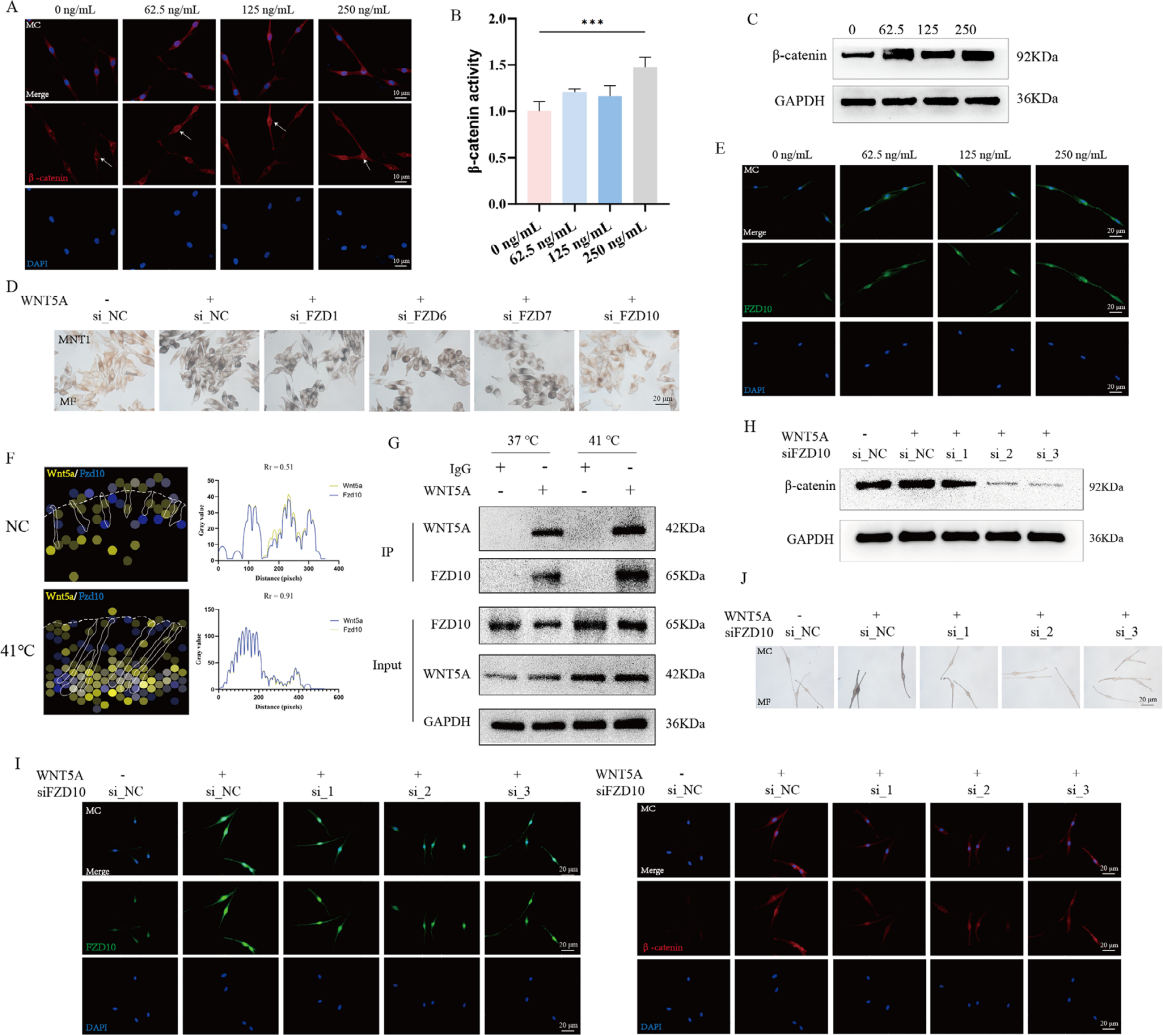
WNT5A activates the FZD10/β‐catenin signaling pathway. (A) Immunofluorescence showing β‐catenin expression in MC cells after 2 days of WNT5A treatment (scale bar = 20 µm). (B) Dual‐luciferase reporter assay of β‐catenin‐dependent TCF/LEF transcriptional activity in MNT1 cells after 2 days of WNT5A treatment. One‐way ANOVA with Tukey's post‐hoc test, F (3, 8) = 12.64, ***p < 0.001. (C) Western blot analysis of β‐catenin protein expression in MNT1 cells after 2 days of WNT5A treatment. (D) Masson‐Fontana staining showing melanin content in MNT1 cells after 2 days of combined treatment with WNT5A and FZD1/6/7/10 knockdown (scale bar = 20 µm). (E) Immunofluorescence showing FZD10 expression in MC cells after 2 days of WNT5A treatment (scale bar = 20 µm). (F) Spatial localization and correlation of WNT5A and FZD10 expression in spatial transcriptomic analysis (Pearson correlation coefficient: 0.5 in the NC group vs. 0.91 in the 41 °C group). (G) Co‐immunoprecipitation (Co‐IP) analysis of the interaction between WNT5A and FZD10 in human skin tissues following heat treatment. (H) Western blot analysis of β‐catenin protein expression in MNT1 cells after 2 days of combined treatment with WNT5A protein and FZD10 knockdown. (I) Immunofluorescence analysis of FZD10 and β‐catenin expression in MC cells after 2 days of combined treatment with WNT5A protein and FZD10 knockdown (scale bar = 20 µm). (J) Masson‐Fontana staining showing melanin content in MC cells after 2 days of combined treatment with WNT5A protein and FZD10 knockdown (scale bar = 20 µm).

To clarify how WNT5A activates β‐catenin, we focused on the WNT pathway receptors we had found to be upregulated at 41°C (FZD1, FZD6, FZD7, and FZD10; Figure 2G). Cellular assays demonstrated that only FZD10 knockdown antagonized the WNT5A‐induced increase in melanin content in MNT1 cells (Figure 4D), indicating that WNT5A promotes melanogenesis primarily through FZD10. We next examined the relationship between WNT5A and FZD10, and found that WNT5A upregulated FZD10 expression (Figure 4E). Moreover spatial transcriptomic analysis revealed substantial overlap in the spatial localization of WNT5A and FZD10, with a markedly enhanced correlation under heat stress (Pearson correlation coefficient: 0.5 in the control group vs. 0.91 in the 41°C group; Figure 4F). Consistently, co‐immunoprecipitation (Co‐IP) assays in cultured human skin tissues confirmed the interaction between WNT5A and FZD10, and this interaction was enhanced under heat stress (Figure 4G). Having validated the WNT5A‐FZD10 interaction and identified FZD10 as the primary receptor mediating WNT5A signaling, we next sought to functionally characterize the role of FZD10 in this axis. We silenced its expression using siRNAs, with efficient knockdown confirmed by qPCR, WB and IF (Figure S5A–C). FZD10 depletion markedly reduced WNT5A‐induced β‐catenin upregulation in both MNT1 (WB) and MC cells (IF) (Figures 4H,I). Consistently, Masson‐Fontana staining showed that FZD10 knockdown significantly impaired WNT5A‐driven melanogenesis in MC cells (Figure 4J).

### 2.5 Heat Stress Induces WNT5A Expression in Fibroblasts

The above experiments established a link between WNT5A and melanogenesis (Figure 3), and spatial transcriptomic analysis confirmed that heat stress induces WNT5A expression (Figure 2). However, the cellular source of WNT5A remained unclear. To address this, we analyzed single‐cell transcriptomic data (GSE150672) and found that WNT5A was highly expressed in fibroblasts, whereas its receptor FZD10 could be expressed in melanocytes (Figure 5A), suggesting that heat stress‐induced WNT5A elevation may originate from fibroblasts. Consistently, FB exposed to heat stress at 39°C and 41°C for 1 h daily over 3 days exhibited significantly increased WNT5A mRNA (Figure 5B) and protein (Figure 5C) levels.

**FIGURE 5:**
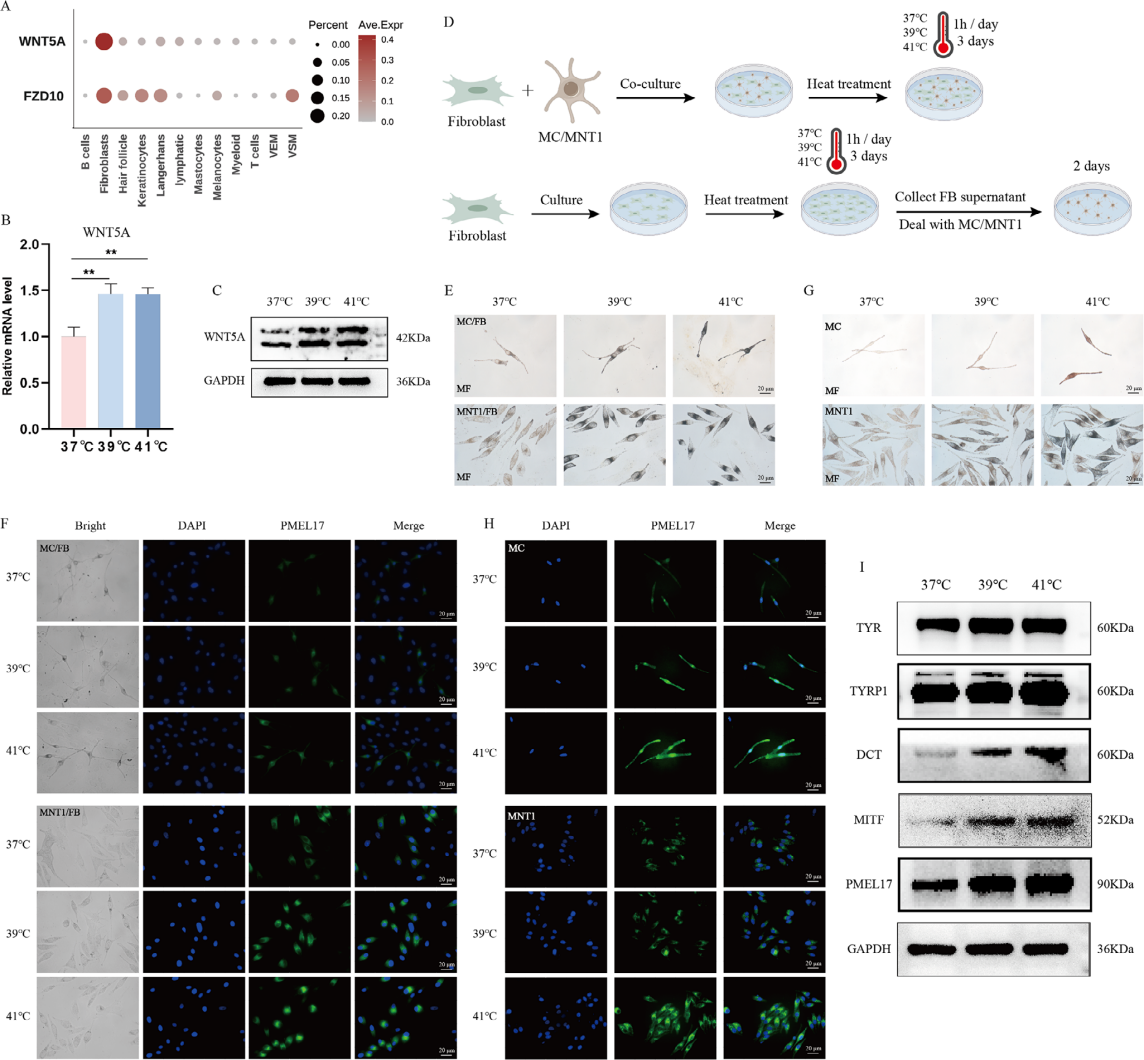
Heat stress induces fibroblast‐derived WNT5A and promotes melanogenesis through fibroblast‐mediated paracrine signaling. (A) Analysis of WNT5A and FZD10 expression across different cell types in the single‐cell dataset (GSE150672). (B) RT‐qPCR analysis of WNT5A mRNA expression in FB cells after heat stress (One‐way ANOVA with Tukey's post‐hoc test, F (2, 6) = 24.21, **p < 0.01). (C) Western blot analysis of WNT5A protein expression in FB cells after heat stress. (D) Schematic diagram of cellular heat treatment procedures. (E) Masson‐Fontana staining was used to assess melanin content in MC and MNT1 cells within the co‐culture system after heat stress (Scale bar = 20 µm). (F) Immunofluorescence localization to observe the fluorescence intensity of PMEL17 in MC and MNT1 cells within the co‐culture system after heat stress (Scale bar = 20 µm). (G) Masson‐Fontana melanin staining to detect melanin content in MC and MNT1 cells after FB supernatant treatment (Scale bar = 20 µm). (H) Immunofluorescence localization to observe the fluorescence intensity of PMEL17 in MC and MNT1 cells after FB supernatant treatment (Scale bar = 20 µm). (I) Western blot analysis of the protein expression levels of melanogenesis‐related regulatory genes in MNT1 cells after FB supernatant treatment.

### 2.6 Heat Stress‐Induced Secretion of WNT5A in Fibroblasts Activates the FZD10/β‐Catenin/Melanogenesis Pathway

Although WNT5A was identified as fibroblast‐derived, the mechanism remains unclear. As WNT5A is a secreted protein, we next sought to determine whether heat stress promotes melanogenesis through fibroblast‐mediated paracrine signaling. MNT1 and FB cells were first exposed to 37, 39, 41, 42, and 43°C for 1 h daily over 3 days (Figure S6A), and cell viability was assessed by CCK‐8. Consistent with previous reports [8], no reduction in viability was observed at 37, 39, or 41°C in either cell type (Figure S6B). Subsequently, we designed a series of cellular heat treatment protocols, including co‐culture and supernatant treatment (Figure 5D). First, we co‐cultured MC or MNT1 cells with FB cells and subjected the co‐culture systems to 37, 39, or 41°C for 1 h daily over 3 days. Masson‐Fontana staining revealed increased melanin content under heat stress (Figure 5E). Immunofluorescence further showed enhanced expression of the melanosome marker PMEL17 in both MC and MNT1 cells (Figure 5F). In addition, TYR probe staining demonstrated increased TYR activity in MNT1 cells, as indicated by elevated yellow fluorescence (Figure S6C).

Then, FB cells were exposed to 37, 39, or 41°C for 1 h daily over 3 days, and supernatants were collected on day 4 to treat MC and MNT1 cells for 2 days. Masson‐Fontana staining revealed increased melanin content in both MC and MNT1 cells following treatment with heat‐stressed FB supernatants (Figure 5G). Immunofluorescence further demonstrated upregulation of the melanosome marker PMEL17 in MC and MNT1 cells (Figure 5H), as well as elevated expression of TYR, TYRP1, and DCT in MNT1 cells (Figure S6D–F). Moreover, WB confirmed that the expression of key melanogenesis regulators (TYR, TYRP1, DCT, MITF, and PMEL17) was elevated in MNT1 cells treated with heat‐stressed FB supernatants (Figure 5I). The above results indicate that heat stress at 39 and 41°C induces FB‐mediated paracrine signaling to promote melanogenesis, with a more pronounced effect observed at 41°C. Given that our earlier findings demonstrated that WNT5A activates the FZD10/β‐catenin signaling pathway (Figure 4) and that heat stress upregulates WNT5A expression in FB cells (Figure 5B,C), we hypothesized that heat stress promotes melanogenesis by stimulating secretion of WNT5A from fibroblasts, which in turn activates FZD10/β‐catenin signaling.

To test this hypothesis, we investigated the role of WNT5A in promoting melanogenesis via the β‐catenin signaling pathway under heat stress. Dual‐luciferase reporter analysis verified that conditioned medium collected from heat‐treated FB promoted β‐catenin‐dependent TCF/LEF transcriptional activity in MNT1 cells (Figure 6A). In MNT1 cells treated with this conditioned medium, WNT5A knockdown decreased β‐catenin protein expression and increased p‐β‐catenin levels (Figure 6B,C), effects that were recapitulated in human skin sections (Figure S6G,H). Furthermore, WNT5A knockdown significantly reduced heat stress‐induced melanin content in both MNT1 cells and human skin sections (Figure 6D,E).

**FIGURE 6:**
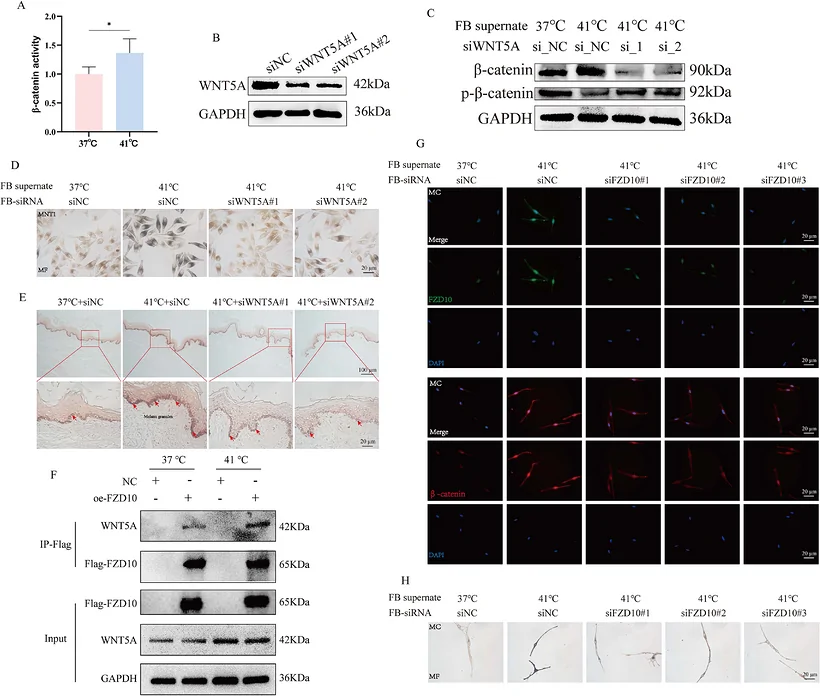
WNT5A secreted from heat‐stressed fibroblasts activates FZD10/β‐catenin signaling to promote melanogenesis. (A) Dual‐luciferase reporter assay of β‐catenin‐dependent TCF/LEF transcriptional activity in MNT1 cells treated with FB‐conditioned medium. Statistical significance was analyzed using Student's t‐test (*p < 0.05). (B) Western blot analysis of WNT5A protein expression in FB cells following WNT5A knockdown. (C) Western blot analysis of the protein expression levels of β‐catenin, p‐β‐catenin in MNT1 cells after 2 days of combined treatment with FB supernatant and WNT5A knockdown. (D) Masson‐Fontana staining showing melanin content in MNT1 cells after 2 days of combined treatment with FB supernatant and WNT5A knockdown (scale bar = 20 µm). (E) Masson‐Fontana staining showing melanin content in human skin sections after 5 days of combined heat treatment and WNT5A knockdown (scale bar = 100 µm, 20 µm). (F) Co‐immunoprecipitation (Co‐IP) analysis of the interaction between WNT5A and FZD10 in MNT1 cells treated with conditioned medium from heat‐treated FB cells. (G) Immunofluorescence analysis of FZD10 and β‐catenin expression in MC cells after 2 days of combined treatment with FB supernatant and FZD10 knockdown (scale bar = 20 µm). (H) Masson‐Fontana staining showing melanin content in MC cells after 2 days of combined treatment with FB supernatant and FZD10 knockdown (scale bar = 20 µm).

Having confirmed the WNT5A–β‐catenin axis under heat stress, we next examined whether FZD10 also mediates this effect. Co‐IP assays performed in MNT1 cells confirmed the interaction between WNT5A and FZD10, and this interaction was enhanced under heat stress conditions (Figure 6F). Immunofluorescence revealed that reduced FZD10 expression attenuated β‐catenin upregulation induced by the heat‐stressed FB supernatants (Figure 6G). Consistently, Masson‐Fontana staining showed that FZD10 knockdown significantly suppressed heat‐stressed FB supernatant‐induced melanogenesis in MC (Figure 6H) and MNT1 (Figure S6I) cells. In contrast, knockdown of other WNT5A receptors, including FZD1, FZD6, and FZD7, failed to suppress melanogenesis induced by heat‐stressed FB supernatants in MNT1 cells (Figure S6J). Collectively, these findings suggest that heat stress enhances melanogenesis by promoting the secretion of WNT5A as a paracrine factor from fibroblasts, which subsequently activates the FZD10/β‐catenin signaling pathway.

### 2.7 Heat Stress Promotes the Expression of WNT5A Through METTL3/YHDC1‐Dependent m^6^A Modification

Heat exposure has been demonstrated to induce epigenetic regulation in organisms [24]. To investigate the potential association between WNT5A expression and epigenetic modifications, we analyzed correlations between WNT5A expression and various epigenetic markers in spatial transcriptomics data. The results revealed significant positive correlations between WNT5A expression and multiple epigenetic modifications, with m^6^A modification showing the strongest association (Figure 7A). Previous studies have also indicated that heat exposure can induce m^6^A modifications [21]. Our data demonstrated a significant upregulation of overall m^6^A modification levels in FB cells following 41°C heat stress (Figure 7B). The m^6^A modification is dynamically regulated by methyltransferases (writers) and demethylases (erasers). The methyltransferase complex core components METTL3, METTL14, and WTAP mediate m^6^A deposition, while FTO and ALKBH5 serves as the primary m^6^A eraser [19]. Initial experiments were conducted to examine the expression patterns of m^6^A writers and erasers in FB cells post‐heat stress. Analysis of the spatial co‐localization between m^6^A regulators (writers and erasers) and WNT5A revealed a pronounced overlap between the spatial domains exhibiting high WNT5A expression and those with elevated METTL3 levels (Figure S7A). Moreover, RT‐qPCR results demonstrated significant upregulation of METTL3 mRNA levels at 0 and 1 h after 41°C heat treatment (Figure 7C). Subsequent WB analysis confirmed that METTL3 protein expression was significantly elevated in FB cells at 2 h after heat stress (Figure 7D). In human skin sections, METTL3 expression was also elevated in Vimentin‐positive fibroblasts after 5 days of heat stress (Figure 7E).

**FIGURE 7:**
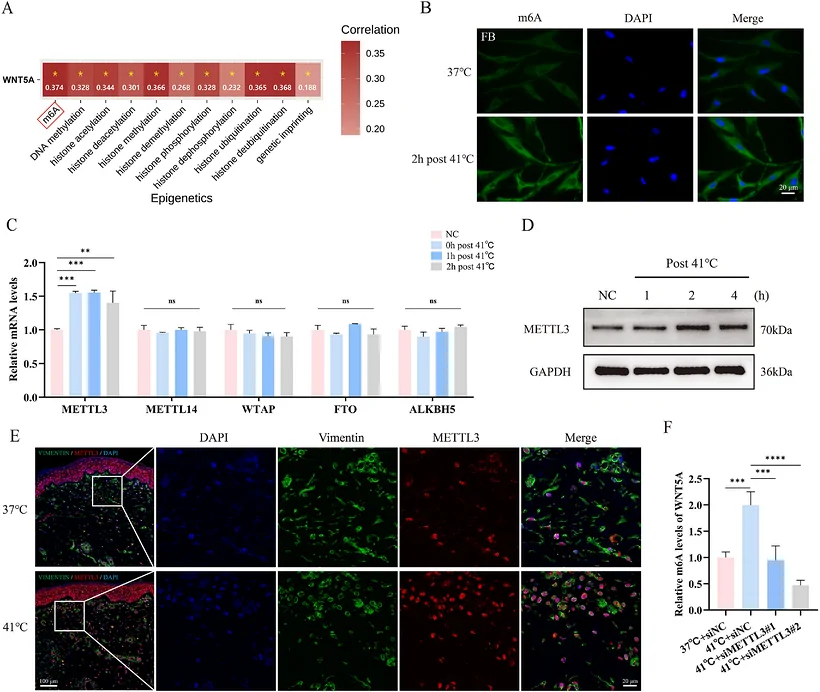
Heat stress promotes melanogenesis by inducing the expression of WNT5A through METTL3/YTHDC1‐dependent m^6^A modification. (A) Correlation analysis between WNT5A expression and epigenetic modifications (Pearson correlation, *p < 0.05). (B) Immunofluorescence analysis of m^6^A modification levels in FB cells 2 h after heat stress (scale bar = 20 µm). (C) RT‐qPCR analysis of METTL3, METTL14, WTAP FTO, and ALKBH5 mRNA expression in FB cells at 0, 1, and 2 h post‐heat stress. One‐way ANOVA followed by Tukey's post‐hoc test. METTL3: F (3, 8) =  24.73, **p < 0.01, ***p < 0.001. No significant differences were found for the other genes. (D) Western blot analysis of METTL3 protein expression in FB cells at 1, 2, and 4 h post‐heat stress. (E) Immunofluorescence images showing METTL3 protein expression (red) in Vimentin‐positive fibroblasts (green) in human skin sections following 5 days of heat stress (scale bar = 100 µm, 20 µm). (F) MeRIP‐qPCR analysis of relative m^6^A levels in WNT5A from heat‐stressed FB cells with METTL3 knockdown. One‐way ANOVA followed by Tukey's post‐hoc test (F (3, 8) =  30.79, ***p < 0.001, ****p < 0.0001).

To further investigate whether METTL3 participates in heat stress‐induced WNT5A in FB cells by m^6^A modification, we predicted potential m^6^A sites on WNT5A mRNA using the SRAMP database and designed primers targeting high‐scoring sites (Figure S7B). MeRIP‐qPCR analysis confirmed that heat stress upregulated m^6^A modification of WNT5A mRNA, and this upregulation was attenuated upon METTL3 knockdown using siRNA in FB cells (Figure 7F and Figure S7C,D). Collectively, these findings establish METTL3 as a critical regulator in heat stress‐mediated m^6^A modification of WNT5A.

Our aforementioned research has shown that heat stress can induce paracrine secretion of WNT5A from FB cells to promote melanogenesis (Figure 3, Figure 5 and Figure 6), and that heat stress increases WNT5A m^6^A modification through a METTL3‐dependent mechanism (Figure 7). Based on these findings, we hypothesized that the melanogenesis‐promoting effect of heat stress‐induced WNT5A upregulation depends on METTL3 activation. STM2457 is a novel and highly selective METTL3 inhibitor suitable for in vivo studies [25]. We first examined the effects of STM2457 on FB cell viability. Results indicated that treatment with 1.25, 2.5, 5, and 10 µM STM2457 for 72 h at either 37 or 41°C had no significant impact on cell viability (Figure S8A). In subsequent experiments, treatment with 2.5 µM STM2457 effectively inhibited the heat stress‐induced elevation of WNT5A mRNA and protein levels in FB cells (Figure 8A and Figure S8B,D), as well as the level of paracrine WNT5A in the supernatant (Figure S8C).

**FIGURE 8:**
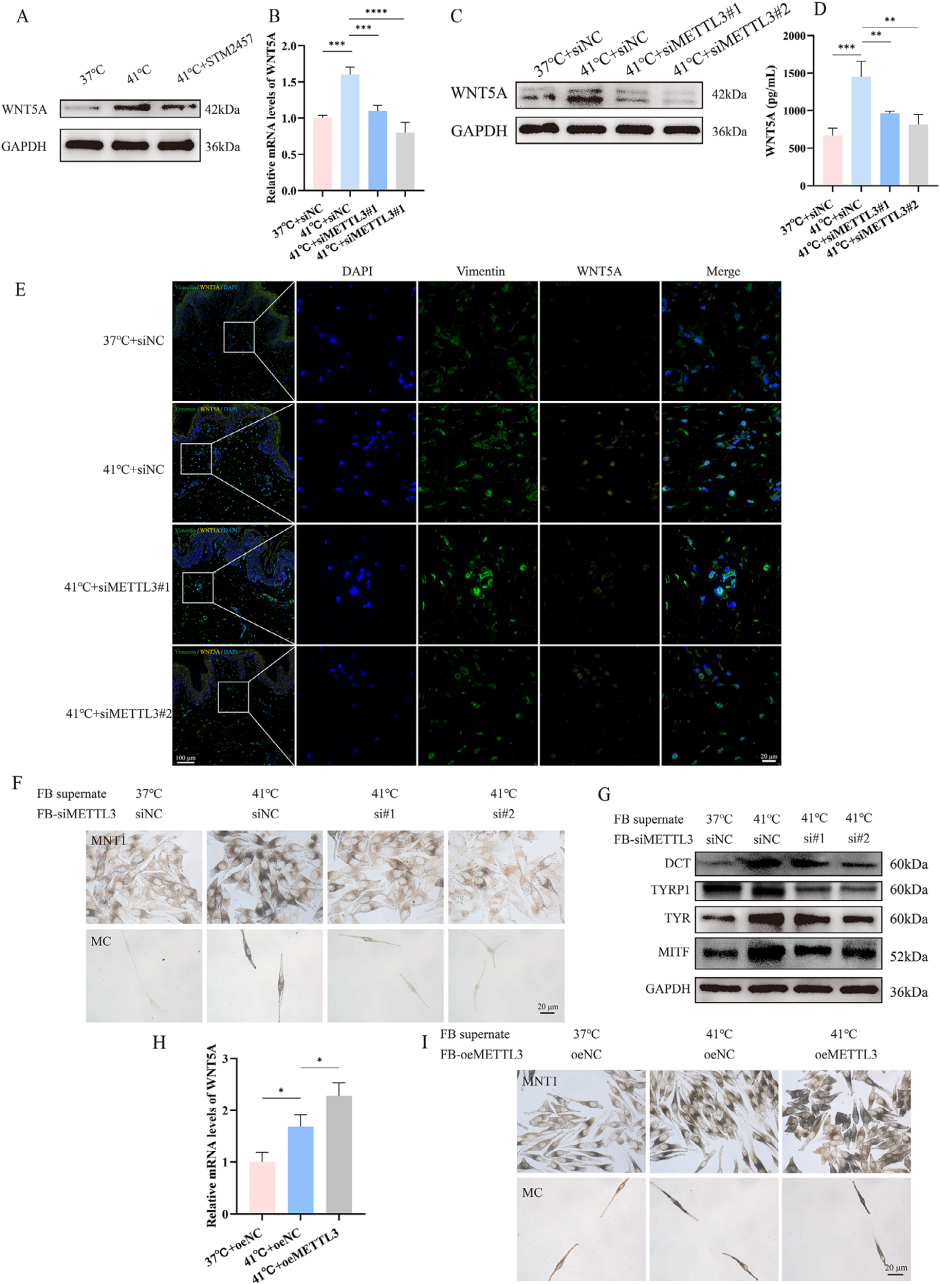
METTL3 mediates heat stress‐induced WNT5A expression and melanogenesis. (A) Western blot analysis of WNT5A protein expression in FB cells pretreated with 2.5 µM STM2457 followed by heat stress. (B) RT‐qPCR analysis of WNT5A mRNA expression in METTL3‐knockdown FB cells following heat stress. One‐way ANOVA followed by Tukey's post‐hoc test (F (3, 8) =  35.08, ***p < 0.001, ****p < 0.0001). (C) Western blot analysis of WNT5A protein expression in FB cells following METTL3 knockdown. (D) ELISA quantification of WNT5A levels in conditioned supernate from METTL3‐knockdown FB cells following heat stress. One‐way ANOVA followed by Tukey's post‐hoc test (F (3, 8) =  19.45, **p < 0.01, ***p < 0.001). (E) Immunofluorescence analysis of WNT5A protein expression (yellow) in vimentin‐positive fibroblasts (green) in human skin sections after 5 days of heat stress with METTL3 knockdown (scale bar = 100 µm, 20 µm). (F) Masson‐Fontana melanin staining of MNT1 and MC cells treated for 2 days with conditioned medium from METTL3‐knockdown FB cells following heat stress (scale bar = 20 µm). (G) Western blot analysis of DCT, TYRP1, TYR, and MITF protein expression in MNT1 cells after treatment with conditioned medium from METTL3‐knockdown fibroblasts. (H) RT‐qPCR analysis of WNT5A mRNA expression in METTL3‐overexpressing FB cells following heat stress. One‐way ANOVA followed by Tukey's post‐hoc test (F (2, 6) =  23.95, *p < 0.05). (I) Masson‐Fontana melanin staining of MNT1 and MC cells treated for 2 days with conditioned medium from METTL3‐overexpressing FB cells following heat stress (scale bar = 20 µm).

To further validate the role of METTL3 in regulating WNT5A expression, we performed functional rescue experiments by knocking down METTL3 in FB cells. The results demonstrated that METTL3 knockdown significantly suppressed the heat stress‐induced upregulation of WNT5A at the mRNA level (Figure 8B), protein level (Figure 8C), and in terms of its paracrine secretion into the supernatant (Figure 8D). Consistent with these cellular findings, METTL3 knockdown also markedly inhibited the heat stress‐induced upregulation of WNT5A expression in human skin sections (Figure 8E).

To examine METTL3's involvement in heat stress‐induced melanogenesis, we collected conditioned medium from STM2457‐treated FB cells and used it to treat MNT1 and MC cells for 2 days. The conditioned medium from heat‐stressed FB cells treated with STM2457 significantly reversed the heat stress‐induced increase in melanin content in both MNT1 and MC cells (Figure S8E). Moreover, METTL3 knockdown significantly reduced heat stress‐induced melanin content in human skin sections (Figure S8F).

Furthermore, when METTL3‐knockdown FB cells were subjected to heat stress and their conditioned medium was applied to MNT1 and MC cells, we observed that METTL3 depletion markedly attenuated the heat stress‐induced increase in melanin content (Figure 8F) and significantly suppressed the upregulation of PMEL17 in both MC cells and MNT1 cells (Figure S8G,H). Moreover, the expression of TYR, TYRP1, DCT were suppressed in MNT1 cells treated with METTL3‐knockdown FB supernatants (Figure 8G). Meanwhile, we also overexpressed METTL3 in FB cells (Figure S8I,J). The results demonstrated that conditioned medium from heat‐stressed, METTL3‐overexpressing FB cells significantly increased WNT5A expression in MNT1 cells (Figure 8H and Figure S8K) and concurrently enhanced the heat stress‐induced melanin increase in both MC and MNT1 cells (Figure 8I). In summary, these findings demonstrate that METTL3 plays a pivotal role in promoting heat stress‐induced WNT5A upregulation, thereby exacerbating heat stress‐stimulated melanogenesis.

In the dynamic regulatory network of m^6^A modification, readers serve as critical executors that connect upstream writing and downstream functional outcomes. They specifically recognize and bind to m^6^A sites to regulate gene expression. To identify key readers involved in heat stress regulated m^6^A modification, we performed RT‐qPCR in FB cells. The results showed that YTHDC1 mRNA levels were significantly upregulated at 0, 1, and 2 h post‐heat stress (Figure 9A). Subsequent experiments confirmed elevated YTHDC1 protein levels at 2 and 4 h (Figure 9B). In human skin sections, YTHDC1 expression was also elevated in Vimentin‐positive fibroblasts after 5 days of heat stress (Figure 9C). To further investigate the role of YTHDC1 in regulating WNT5A expression, we performed RIP‐qPCR and RNA pull‐down assays, which confirmed a direct interaction between the YTHDC1 protein and WNT5A mRNA (Figure S9A,B). Subsequently, we knocked down YTHDC1 in fibroblasts using siRNA (Figure 9D). To validate the specificity of the siRNA‐mediated knockdown, fibroblasts were subjected to YTHDC1 knockdown followed by heat stress. Western blot analysis demonstrated that siRNA efficiently suppressed the heat stress‐induced elevation of YTHDC1 protein levels, while having no effect on YTHDC2 protein expression, confirming the specificity of the siRNA (Figure S9C). Further experiments revealed that YTHDC1 knockdown suppressed both mRNA and protein expression of WNT5A in heat‐stressed FB cells (Figure 9E,F). Given that prior literature highlights YTHDC1's role in regulating mRNA stability [26, 27], we evaluated WNT5A mRNA stability following heat stress with concurrent YTHDC1 knockdown. By measuring WNT5A mRNA levels after actinomycin D treatment, we found a shorter half‐life of WNT5A mRNA in siYTHDC1 knockdown cells (Figure 9G). Furthermore, application of conditioned medium from heat‐stressed FB cells to MNT1 cells revealed that YTHDC1 knockdown significantly attenuated heat stress‐induced melanogenesis in both MC and MNT1 cells (Figure 9H). This was accompanied by a marked reduction in the expression levels of melanogenesis‐associated proteins (DCT, TYR, and MITF) in MNT1 cells (Figure S9D), as well as suppression of PMEL17 upregulation in both MC and MNT1 cells (Figure S9E,F). Moreover, human skin sections with YTHDC1 knockdown were exposed to heat stress for five days. We similarly found that YTHDC1 knockdown antagonized the heat stress‐induced upregulation of WNT5A expression and melanin content in human skin tissue (Figure S9G,H). These findings confirm that YTHDC1 is required for heat‑induced melanogenesis in human skin. In summary, these findings indicate that YTHDC1 plays a crucial role in heat stress‐induced upregulation of WNT5A by regulating its mRNA stability, thereby exacerbating heat stress‐stimulated melanogenesis.

**FIGURE 9:**
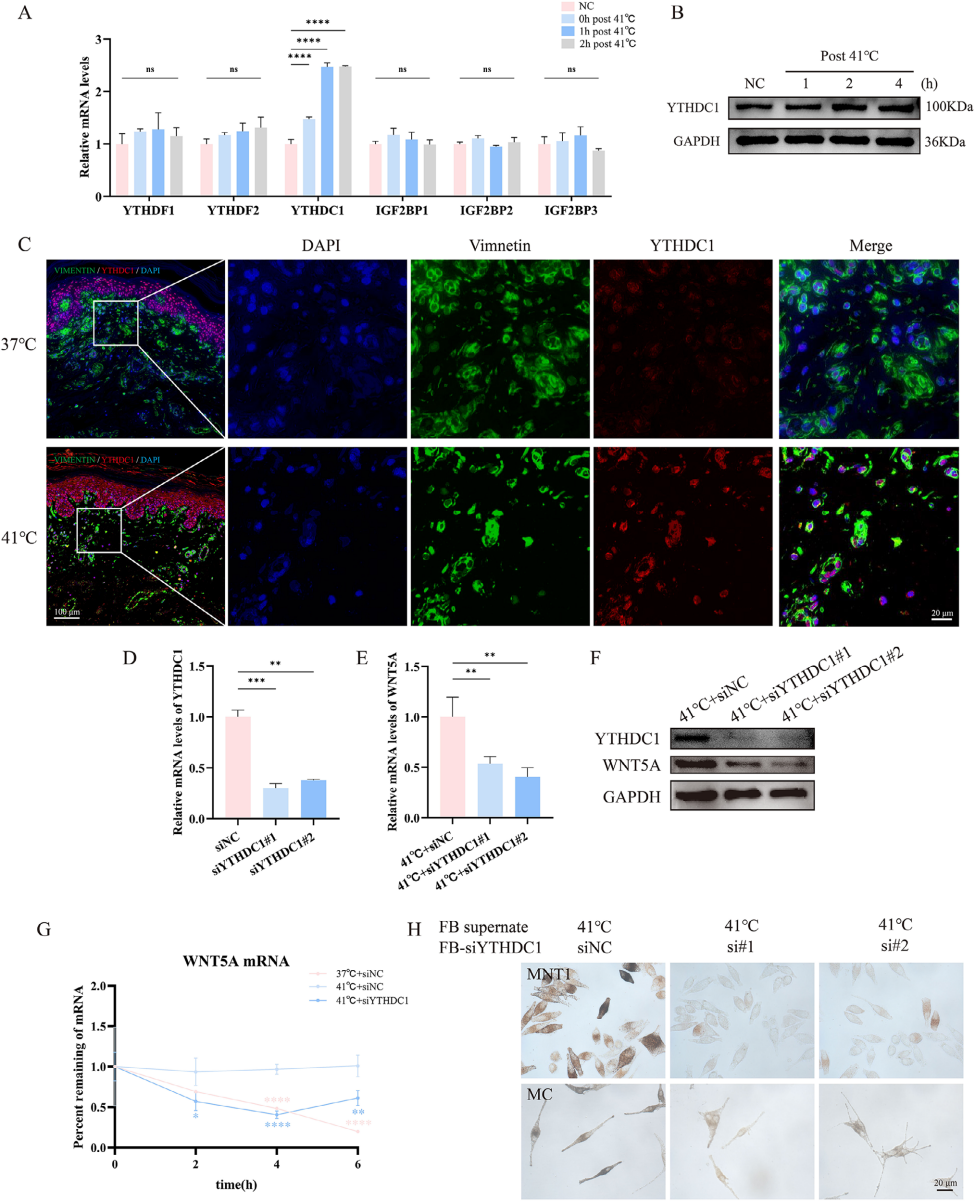
YTHDC1 mediates heat stress‐induced WNT5A expression and melanogenesis in fibroblasts. (A) RT‐qPCR analysis of YTHDF1, YTHDF2, YTHDC1, IGF2BP1, IGF2BP2, and IGF2BP3 mRNA expression in FB cells at 0, 1, and 2 h post‐heat stress. One‐way ANOVA followed by Tukey's post‐hoc test. YTHDC1: F (3, 8) =  415, ****p < 0.0001. No significant differences were found for the other genes. (B) Western blot analysis of YTHDC1 protein expression in FB cells at 0, 1, 2, and 4 h post‐heat stress. (C) Immunofluorescence images showing YTHDC1 protein expression in Vimentin‐positive fibroblasts in human skin sections following 5 days of heat stress (scale bar = 100 µm, 20 µm). (D) RT‐qPCR analysis of YTHDC1 mRNA expression in FB cells following YTHDC1 knockdown. One‐way ANOVA followed by Tukey's post‐hoc test (F (2, 6) =  195.3, **p < 0.01, ***p < 0.001). (E) RT‐qPCR analysis of WNT5A mRNA expression in YTHDC1‐knockdown FB cells following heat stress. One‐way ANOVA followed by Tukey's post‐hoc test (F (2, 6) =  17.02, **p < 0.01). (F) Western blot analysis of YTHDC1 and WNT5A protein expression in FB cells following YTHDC1 knockdown. (G) RT‐qPCR analysis of WNT5A mRNA in YTHDC1‐knockdown FB cells after actinomycin D treatment for 0, 2, 4, and 6 h. One‐way ANOVA followed by Tukey's post‐hoc test (0h: no significant differences; 2h: F (2, 6) =  5.859, *p < 0.05; 4h: F (2, 6) =  125.4, ****p < 0.0001, 6h: F (2, 6) =  54.57, **p < 0.01, ****p < 0.0001). (H) Masson‐Fontana staining of MC and MNT1 cells treated for 2 days with conditioned medium from YTHDC1‐knockdown FB cells following heat stress (scale bar = 20 µm).

## 3 Discussion

This study demonstrates that heat stress promotes the secretion of WNT5A from fibroblasts through METTL3/YTHDC1‑dependent m^6^A methylation modification. WNT5A binds to the FZD10 receptor on the membrane of melanocytes, thereby inhibiting the phosphorylation‑dependent degradation of β‑catenin. The accumulated β‑catenin in melanocytes is then translocated into the nucleus, where it drives the expression of key melanogenesis‑related genes, ultimately promoting melanin synthesis. Collectively, this study reveals that heat stress regulates melanogenesis through the m^6^A‑modified WNT5A/FZD10/β‑catenin signaling pathway (Figure 10).

**FIGURE 10:**
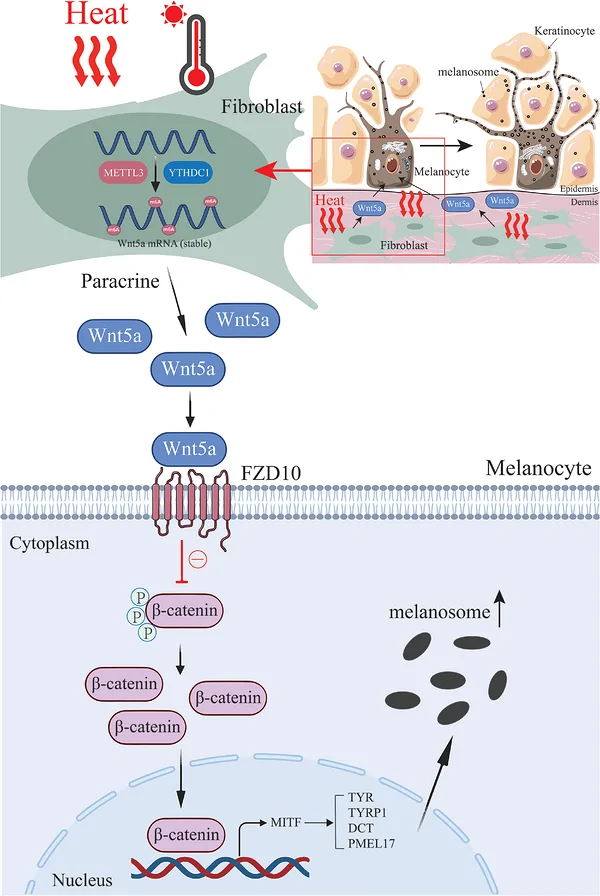
Mechanism diagram illustrating heat stress promotes melanogenesis via m^6^A modification‐enhanced fibroblast secretion of WNT5A. Heat stress regulates the stability of WNT5A mRNA through METTL3/YTHDC1‐dependent m^6^A modification in fibroblasts, thereby promoting the paracrine secretion of WNT5A. In melanocytes, WNT5A drives melanogenesis by activating the FZD10/β‐catenin signaling pathway.

Previous studies have demonstrated that heat and UVB exert comparable biological effects on melanocytes, with heat alone being sufficient to directly modulate melanogenesis [8]. Cutaneous pigmentation is orchestrated by a complex dermal‐epidermal network, wherein fibroblast‐derived factors interact with melanocyte receptors to regulate intracellular melanogenic pathways [28]. Our earlier work revealed that heat stress enhances keratinocyte paracrine activity via activation of the TRPV3/Ca^2^^+^/Hh signaling axis, thereby stimulating melanogenesis [8]. Additionally, we found that heat stress induces melanogenesis via the activation of the CX3CL1/CX3CR1 axis through WDR5‐mediated H3K4me3 modification [7]. However, no previous studies have explored how heat stress‐induced fibroblast paracrine signaling affects melanogenesis. The present study therefore fills this important research gap. Our findings indicate that the m^6^A‐WNT5A pathway‐dependent paracrine effect in fibroblast represents an additional independent mechanism regulating melanin production under heat stress.

The Wnt signaling pathway plays a pivotal role in melanocyte differentiation [29, 30]. In particular, WNT3 and WNT1 act as canonical ligands that activate the Wnt/β‐catenin pathway, driving the expression of melanogenesis‐related genes [31, 32]. Mechanistically, Wnt proteins engage G‐protein‐coupled receptors of the Frizzled (FZD) family, inhibiting β‐catenin phosphorylation and subsequent degradation, thereby enabling β‐catenin stabilization, cytoplasmic accumulation, and nuclear translocation [13]. In the nucleus, β‐catenin enhances MITF transcription, ultimately promoting melanocyte survival, proliferation, and melanin synthesis [12, 13].

Our findings establish a strong link between WNT5A and melanogenesis. WNT5A upregulated FZD10 expression, and functional rescue experiments demonstrated that knockdown of FZD10 attenuated WNT5A‐induced β‐catenin activation and melanin production, confirming that FZD10 serves as a WNT5A receptor in this context. Collectively, these results indicate that WNT5A promotes melanogenesis through the FZD10/β‐catenin pathway.

Spatial transcriptomic analyses revealed heat‐induced upregulation of WNT5A, while single‐cell transcriptomic data (GSE150672) indicated fibroblasts as the primary source of WNT5A. Consistently, molecular assays confirmed that heat stress induced WNT5A expression at both mRNA and protein levels in fibroblasts. Subsequent co‐culture and conditioned media experiments further validated the paracrine role of fibroblasts in mediating heat‐induced melanogenesis. Importantly, prior clinical studies have shown elevated WNT5A expression in melasma lesions, a condition closely associated with thermal triggers [16], consistent with our observations. Finally, functional rescue experiments confirmed the involvement of FZD10 in heat‐induced melanogenesis: silencing of FZD10 markedly attenuated β‐catenin activation and melanin accumulation. Taken together, these results demonstrate that heat stress stimulates fibroblasts to secrete WNT5A, which in turn activates the FZD10/β‐catenin signaling cascade to promote melanogenesis.

m^6^A modification, the most prevalent epigenetic RNA modification in eukaryotic cells, plays a pivotal role in determining the fate of modified RNA molecules at the post‐transcriptional level, influencing nearly all critical biological processes [33]. Emerging evidence indicates that m^6^A methylation participates in the regulation of heat stress responses [21, 22, 23, 34]. As the core catalytic component of the m^6^A methyltransferase complex, METTL3 has been shown to be functionally significant during heat stress. Previous studies reported that heat stress (42°C) markedly upregulated METTL3 levels in primary hepatocytes and preadipocytes of Hu sheep [35, 36]. Similarly, 44°C heat stress was found to elevate METTL3 expression in porcine Sertoli cells [23]. In line with these findings, our study demonstrates that cutaneous fibroblasts subjected to 41°C heat stress exhibit a comparable increase in METTL3 expression. Notably, METTL3‐dependent m^6^A methylation has been implicated in the heat‐induced upregulation of heat shock proteins (HSPs), suggesting that many other heat‐responsive genes may be regulated through a similar mechanism [22]. In our study, we found that heat stress treatment increased the m^6^A modification level of WNT5A mRNA, which subsequently led to a significant upregulation of its expression. Notably, this regulatory process was primarily mediated by METTL3.

As a crucial nuclear m^6^A reader, YTHDC1 has been demonstrated to play an important functional role during heat stress. Previous research reported that YTHDC1 promotes the co‐transcriptional induction of heat shock proteins in a METTL3‐dependent manner under heat stress [22]. Furthermore, genomic analysis of Sadah sheep raised under different temperatures suggested that YTHDC1 may be indirectly associated with ovine thermotolerance [37]. Similarly, another study found that knockdown of YTHDC1 impairs the regulatory capacity of Drosophila in response to heat stress [38]. Consistent with these findings, our study shows that the expression of YTHDC1 is increased in fibroblasts subjected to 41°C heat stress. By regulating the stability of WNT5A mRNA, YTHDC1 mediates the heat stress‐induced upregulation of WNT5A, which subsequently promotes melanogenesis in melanocytes.

Our study originally demonstrates that heat stress promotes melanogenesis by enhancing the paracrine secretion of WNT5A from fibroblasts via m^6^A modification. These findings provide novel mechanistic insights into the pathogenesis of heat‐induced pigmentation disorders, including melasma, erythema ab igne, and post‑laser hyperpigmentation. Our study provides several insights with potential translational value. First, it establishes a direct mechanistic link between heat stress and melanogenesis via m^6^A‐mediated WNT5A secretion from dermal fibroblasts. Second, the pro‐pigmentary effect of heat stimulation raises the possibility of thermotherapy as a novel, non‐UV intervention for hypopigmentary disorders such as vitiligo. Unlike phototherapy, local heat application may offer a lower risk of photocarcinogenesis and could be combined with existing treatments. Third, the identification of WNT5A and FZD10 as essential mediators suggests that pharmacological activation of this axis—for example, with WNT5A mimetics or small‐molecule FZD10 agonists—might represent a targeted strategy to stimulate repigmentation.

Nevertheless, similar to the double‐edged sword effect of UV radiation in vitiligo treatment—where specific wavelengths serve as a primary modality in phototherapy, while photodamage may potentially trigger koebner phenomenon [39, 40]—excessive or prolonged thermal exposure may induce koebner phenomenon in vitiligo [41]. Therefore, when applying heat therapy for the treatment of hypopigmented lesions such as vitiligo, potential therapeutic risks should be carefully considered. Excessive treatment duration or excessively high temperatures may lead to local adverse effects, including erythema, blister formation, ulceration, and secondary infection. In addition, prolonged or excessive heat exposure may also cause hyperpigmentation. Therefore, to ensure both the efficacy of promoting repigmentation in depigmented vitiligo lesions and the minimization of potential side effects, it is essential to strictly control both the temperature and the duration of heat therapy.

In conclusion, this study further reveals that heat stress may promote the secretion of WNT5A from fibroblasts through METTL3/YTHDC1‐dependent m^6^A modification, and activate the FZD10/β‐catenin signaling pathway in melanocytes to drive melanogenesis. These findings provide new insights and potential molecular targets for the clinical prevention and treatment of pigmentary skin disorders, including both hyperpigmented conditions (such as melasma and erythema ab igne) and hypopigmented conditions (such as vitiligo).

## 4 Materials and Methods

### 4.1 Thermotherapy

This study was approved by the Third Xiangya Hospital of Central South University ethics committee (NO.24385). The lesions of patients with vitiligo were divided into two groups. The control group received conventional therapy, which involved phototherapy, along with corticosteroids, calcineurin inhibitors, or CO2 laser, etc. On the basis of conventional therapy, the thermotherapy group received an additional local heating. The warmer pads (wecan, China) were used for heating on the lesions once a day for 20 min each time due to their wide accessibility and user‐friendliness. After tearing open the outer protective bag, the iron powder in the warmer pad quickly oxidizes and releases heat upon contact with the air, raising the surface temperature to about 41°C. The area of lesions was measured before and after the treatment. The repigmentation of lesions was compared between the two groups after 12 weeks of treatment. The informed consent template is shown in Table S6.

### 4.2 Cell Culture

The human melanoma cell line MNT1 (Meisen CTCC, China, #CTCC‐006‐0301) cells were cultured in DMEM medium (Gibco, USA, #C11995500BT) supplemented with 20% fetal bovine serum (FBS) (Meisen CTCC, China, #CTCC‐002‐071), 1% penicillin‐streptomycin (G‐CLONE, China, #ABT920) and 1% Nonessential Amino Acids (Meisen CTCC, China, #CTCC‐002‐142).

After approval by the Ethics Committee of the Third Xiangya Hospital of Central South University (No.24910), the human epidermal melanocytes (MC) and human dermal fibroblasts (FB) were extracted from adolescent foreskin tissues donated after circumcision. MC cells were cultured in 254 medium (Gibco, USA, #M254500) with 1% HMGS (Gibco, USA, #S0025) and 1% penicillin‐streptomycin. FB cells were cultured in DMEM medium supplemented with 10% FBS (Viva Cell, China, #C04001‐500), 1% penicillin‐streptomycin. None of the cell lines were detected to be contaminated with mycoplasma. Cells were cultured in a humidified incubator at 37°C and 5% CO_2_. To inhibit METTL3, FB cells were treated with STM2457(MedChemExpress, USA, #HY‐134836) dissolved in DMSO (MP Biomedicals, USA, #196055) for 4 consecutive days.

### 4.3 Extraction of Primary Cells

The foreskin tissue donated by adolescent patients undergoing circumcision at the Third Xiangya Hospital of Central South University (with the approval of both the donors and the Ethics Committee of the Third Xiangya Hospital, Central South University) was collected and immersed in dulbecco's phosphate‐buffered saline (D‐PBS) buffer containing 10% penicillin‐streptomycin‐amphotericin B solution and stored in a 4°C refrigerator for 4 h. Then, the foreskin tissue was washed 3–5 times with D‐PBS solution. The foreskin tissue was placed in a sterile culture dish with the epidermal side facing down, and subcutaneous tissue was removed using surgical scissors. The foreskin tissue was cut into circular pieces approximately the size of a coin using surgical scissors and soaked in a culture dish containing center‐proteinase II (2 mg mL^−1^) (Solarbio, China, #D6430) with the epidermal side facing up and the dermal side facing down. The culture dish was then refrigerated overnight at 4°C. Then, the skin pieces were gently rinsed 2–3 times using D‐PBS solution. The dermal layer of the skin pieces was pressed downward with forceps held in the left hand, while the epidermal layer was lifted gently with forceps held in the right hand to separate the epidermis from the dermis.

Melanocytes: The separated epidermal tissue was placed in a culture dish and rinsed 1–2 times with D‐PBS solution. The cleaned skin pieces were transferred to a sterile culture dish and minced using ophthalmic scissors. Subsequently, 5 mL of a 0.25% trypsin solution (Beyotime, China, #C0201) was added and transferred to a centrifuge tube and digested in a 37°C incubator for 10 min. Finally, the cell suspension was filtered through a cell strainer to obtain the filtered cell suspension. The filtered cells were washed twice with D‐PBS solution. Ultimately, the cells were resuspended in MC medium.

Fibroblasts: The separated dermal tissue was placed in a culture dish and rinsed 1–2 times with D‐PBS solution. The cleaned skin pieces were transferred to a sterile culture dish and minced using ophthalmic scissors. The minced dermal tissues were spread evenly in sterile culture dishes and dry it in a 37°C incubator for 4 h. After 4 h, add FB medium to the culture dish and continue to culture it in the cell incubator.

### 4.4 Heat Treatment of Cells

Cells were inoculated into the petri dishes at an appropriate density. According to our previous study [8], after the cells attached, the petri dishes were placed in a high temperature incubator with a constant temperature of 37, 39, or 41°C for hear treatment for 1 h and then the petri dishes were placed back in a constant temperature incubator at 37°C for further culture, and the above steps were repeated for 3 consecutive days. Meanwhile, the O_2_ and CO_2_ concentrations in the constant temperature incubator were measured at 0, 15, 30, 45, and 60 min during cell heat treatment.

### 4.5 Cell Transfection

Cell transfection was performed using the CALNP RNAi in vitro transfection reagent (D‐Nano Therapeutics,China, #DN001‐ 01) according to the manufacturer's instructions. Briefly, cells were digested, centrifuged, and resuspended, then evenly seeded into 6‐well or 12‐well plates and incubated in a culture incubator. When the cell confluency reached 60% – 70%, transfection was carried out following the manufacturer's protocol. The sequences of siRNAs are presented in Table S**3**.

### 4.6 Human Skin Culture and Interventions

Human skin samples were obtained from healthy adolescent foreskins after circumcision, following approval by donors and the Ethics Committee of the Third Xiangya Hospital of Central South University. Briefly, subcutaneous tissue and fat were removed from the foreskin samples, and the latter were cut into 2 cm^2^ pieces. For WNT5A treatment, the skin pieces were placed on DMEM included with 10% FBS, 1% penicillin‐streptomycin‐amphotericin B and WNT5A (0, 62.5, 125, 250 ng mL^−1^) with the epidermis side up. The plates were incubated at 37°C with 5% CO_2_ for 5 days. Moreover, for heat intervention and siRNA knockdown: the skin tissues were cultured continuously for 5 days and exposed to 41°C for 1 h daily. And on days 1 and 3 of the culture period, 30 µL (2.5 nmol) of siRNA solutions targeting WNT5A, YTHDC1, or METTL3 were locally injected at a deep depth between the dermis and epidermis.

### 4.7 Constructing a Depigmented Mouse Model

Five‐week‐old C57BL/6J mice (half male and half female) were provided by Hunan Slaike Jingda Laboratory Animal Co. LTD. All animal experimental protocols were approved by the Animal Welfare Committee (Ethics approval number: CSU‐2022‐0525). After one week of acclimatization feeding, the dorsal hair of the mice was shaved. The mice were divided into two groups: normal group and depigmentation model group. Mice in the depigmentation model group were treated with 40% monobenzone cream (Briton, India) twice daily, while those in the normal group received Vaseline twice daily as a control. The treatments were continued for 6 weeks.

### 4.8 Heat Treatment of Mice

Upon the successful induction of a depigmented model in mice, the animals were systematically allocated into two distinct groups, each consisting of five mice: negative control (NC) group and heat treatment (41°C) group. The NC group was left untreated to serve as a baseline reference. For the (41°C) group, a 41°C heating pad (Warm U, China) was securely affixed to the backs of the mice, delivering a daily 30 min session of warmth. This regimen was maintained for a total of 5 weeks, during which the therapeutic outcomes were meticulously monitored and documented.

In addition, six healthy non‐modeled C57BL/6J mice were randomly and equally assigned to the negative control (NC) group and heat treatment (41°C) group, with 3 mice per group. During the daily 30 min heat exposure session, the dorsal skin surface temperature of the heated region and rectal core temperature were measured in mice of the heat treatment group at 0, 10, 20, and 30 min. Rectal temperature was detected using a digital thermometer dedicated to laboratory animals(TH212, Beijing Shanshui Xinke Testing Technology Co., Ltd.,China), while dorsal skin temperature was recorded via an infrared thermal imager(testo 871, Testo SE & Co. KgaA, Germany). Meanwhile, peripheral blood samples were collected from mice in both groups at day 1, day 3 and day 7 after the initial heat exposure to examine serum corticosterone levels.

### 4.9 Masson‐Fontana Melanin Staining

Masson‐Fontana melanin staining, which utilizes an ammoniacal silver solution for the detection of melanin granules, represents the most commonly employed method for melanin staining [42].

*Cells*: When the cell confluence in the well plate reached 70%–80%, the culture medium was discarded and the cells were washed thrice with PBS buffer. The cells were fixed for 15–20 min with a 4% paraformaldehyde fixative, then the paraformaldehyde solution was discarded and the cells were washed three times with double‐distilled water, each for 5 min. 1000 µL of ammoniacal silver solution was added to each well of the well plate and incubated in a 56°C water bath, shielded from light, for 5–15 min. Subsequently, 1 mL of sodium thiosulfate solution was added and allowed to react for 3–5 min. The sodium thiosulfate solution was discarded, the cells were washed with double‐distilled water three times, and finally, 1 mL of PBS buffer was added to each well.

*Tissue Sections*: Tissue sections were dried in an oven at 60°C for 30 min, then subjected to deparaffinization, xylene clearing, and ethanol‐based hydration, culminating in a tap water rinse. Subsequently, the sections were positioned flat in a dark container, treated with 50 µL of ammoniacal silver solution, and incubated at 56°C in a light‐protected water bath for 30 min. Following triple washing with double‐distilled water, 50 µL of sodium thiosulfate solution was applied for 3–5 min, followed by an additional three washes. The sections were then treated with 50 µL of neutral red staining solution for 2–3 min, washed three more times, air‐dried, and mounted.

Images of cells and tissue sections were acquired using a ZEISS inverted fluorescence microscope (LSM800). For each batch of samples, consistent exposure time and white balance were maintained during image acquisition. The integrated optical density (IOD) of Masson‐Fontana‐positive signals within the hair follicle areas was measured using ImageJ software (version 1.54, USA) with a fixed threshold and normalized to the total epidermal area, reflecting the melanin content per unit epidermal area.

### 4.10 Methylated RNA Immunoprecipitation (MeRIP)

Total RNA was extracted using the described method, and the m^6^A RNA fraction was enriched using the EpiQuik CUT&RUN m^6^A RNA Enrichment (MeRIP) Kit (EpigenTek, USA, #P‐9018‐24) [43, 44]. For each sample, 10 µL of the enriched RNA was subjected to reverse transcription and amplification. The primers are shown in Table S4.

### 4.11 ELISA

Serum corticosterone levels were measured using a commercial ELISA kit (Cloud‐Clone Corp, China; Cat. No. CEA540Ge), and WNT5A levels in cell culture supernatants were measured using another ELISA kit (Mlbio, China; Cat. No. ml1005067), both according to the manufacturer's protocols. A standard curve was generated using two‐fold serial dilutions of the provided standard, ranging from 0.16 to 10 ng mL^−1^, with a blank well containing only standard diluent (0 ng mL^−1^). According to the manufacturer, the intra‐assay precision (coefficient of variation, CV) was < 8% (three known samples assayed 20 times on a single plate), and the inter‐assay precision (CV) was < 10% (three known samples assayed 40 times across three independent plates). Supernatants were assayed undiluted, and all samples were measured in duplicate.

### 4.12 Dual‐Luciferase Reporter Assay for WNT/β‐Catenin Signalling

MNT1 cells were co‐transfected with the TOPFlash (Beyotime, China, D2503) and pRL‐TK (Beyotime, China, #D2760) reporter plasmids at a ratio of 15:1. After transfection, cells were treated with the indicated drugs. Luciferase activities were measured using a dual‐luciferase reporter assay kit according to the manufacturer's instructions (Beyotime, China, RG027). Briefly, cells were lysed, and the lysates were centrifuged. The supernatants were sequentially assayed for firefly and then Renilla luciferase activity using a microplate reader. The ratio of firefly to Renilla luminescence was calculated to normalize for transfection efficiency and cell number, and used to compare the relative activation level of the WNT/β‐catenin pathway across groups.

### 4.13 RNA Immunoprecipitation (RIP)‐qPCR

To explore the interaction between YTHDC1 protein and WNT5A mRNA, we performed RIP experiments according to the manufacturer's instructions (PureBinding RNA Immunoprecipitation Kit, China, #P0101). For each RIP reaction, 10^7^ FB cells were collected and washed with PBS. Then the pellet was resuspended in a buffer containing protease inhibitors and ribonuclease (RNase) inhibitors. The resulting cell lysate was divided into three groups (input group, immunoprecipitation group, and immunoglobulin G (IgG) control group). Magnetic bead protein A/G was incubated with 5 µg of YTHDC1 antibody and IgG antibody respectively at 4°C for 1 h. Subsequently, the YTHDC1 antibody and IgG antibody conjugates were incubated with the cell lysates of the immunoprecipitation group and the IgG group respectively at 4°C for 2 h. Finally, the protein‐bound RNA was extracted according to the manufacturer's protocol. The relative abundance of protein‐bound RNA was determined by RT‐qPCR analysis.

### 4.14 RNA Pulldown Assay

RNA pulldown was performed using a commercial kit (Tsingke, China, DLI201) following the manufacturer's instructions. Biotin‐labeled RNA probes (WNT5A mRNA or negative control) were folded into secondary structures. Streptavidin magnetic beads were washed, mixed with the folded probes, incubated at room temperature, and resuspended in binding buffer. Cells were lysed in pulldown buffer containing protease and RNase inhibitors, sonicated on ice, and centrifuged. A portion of the supernatant was saved as input. The remaining supernatant was combined with the probe‐conjugated beads, enhancer, and additional pulldown buffer, then incubated with rotation. Beads were captured and washed three times. Bound proteins were eluted in elution buffer at 65 °C for western blotting.

### 4.15 Actinomycin D Treatment

Actinomycin D (MedChemExpress, USA, #HY‐17559) was dissolved in DMSO to prepare a stock solution at 5 mg mL^−1^. FB cells were then treated with actinomycin D at a working concentration of 5 µg mL^−1^ (1:1000 dilution from the stock) to inhibit mRNA transcription. Cells were harvested at 0, 4, and 6 h post‐treatment. Total RNA was extracted and subjected to RT‐qPCR analysis.

### 4.16 Spatial Transcriptomics Preparation and Sequencing

#### 4.16.1 Sample Preparation

Spatial transcriptomics data were obtained by Shanghai Biotechnology Corporation (SHBIO, Shanghai, China). Spatial transcriptomic profiling was conducted using the 10x Genomics Visium CytAssist platform. We collected skin tissue samples from mice in both the negative control (NC) and heat treatment (41°C) groups, followed by formalin fixation and paraffin embedding (FFPE).

The tissue was carefully mounted onto Visium CytAssist Gene Expression Slides and underwent a series of procedures, including deparaffinization, staining, imaging, decrosslinking and probe hybridization, with the aim of capturing spatially resolved mRNA transcripts. Subsequently, the Visium CytAssist gene expression chips were inserted into the Visium CytAssist instrument for bright‐field imaging. Within the instrument, analyte transfer was performed, allowing precise capture of transcriptomic information from the FFPE tissue. Raw sequencing data were processed using the Space Ranger pipeline (v.2.1.1).

#### 4.16.2 Data Quality Control and Processing

For spatial sequencing data, raw UMI count spot matrices, images and spot‐image coordinates were imported into R (version 4.3.0). The expression profile, sample information (metadata), and H&E staining images (spatial data only) were integrated into a Seurat object by R package Seurat (version 4.9.9). Spot matrix was filtered out to keep only spots overlaying tissue sections. Raw UMI counts were normalized using regularized negative binomial regression (SC Transform). After identifying the 2000 most variable genes (FindVariableFeatures), dimensionality reduction was performed using Principal Component Analysis (PCA) and t‐Distributed Stochastic Neighbor Embedding (t‐SNE). Significantly differentially expressed genes (DEGs) were screened based on *p*‐values (*p* < 0.05) and log2foldchange (absolute values in the top 5%). DEGs were visualized using a volcano plot, and their functional annotations and interpretations were performed using GO and KEGG enrichment analysis.

### 4.17 RNA and Single‐Cell Transcriptomics Sequencing

#### 4.17.1 Datasets Collection

The public datasets used in this work were downloaded from GEO (http://www.ncbi.nlm.nih.gov/geo/). One dataset each of RNA high‐throughput sequencing of melasma (GSE72140) and human skin high‐throughput single‐cell data (GSE150672)were selected as the validation set (Table S5). Data was processed by R (version 4.3.0) on Linux system (Ubuntu, version 20.04).

#### 4.17.2 Dataset Processing and Enrichment Analysis

Using limma package (3.46.0) to analyze differentially expressed genes (DEGs) between the melasma group and the normal group in the GSE72140 dataset, and DEGs with statistical significance were selected based on *p*‐values (*p* < 0.05) and logFC. DEGs are displayed using a volcano plot, and the expression of WNT5A and melanogenesis‐related regulatory genes between the two groups was displayed using a violin plot. Differentially expressed genes were ranked according to the absolute value of logFC, and the top 300 significantly upregulated genes with *p*‐values < 0.05 were selected for GO and KEGG enrichment analysis. Subsequently, based on the average expression levels of TYR in each sample, the melasma samples were divided into high‐expression and low‐expression groups of TYR. Analyze DEGs between the two groups, and the up‐regulated DEGs were subjected to GO and KEGG enrichment analysis. Expression of WNT5A and FZD10 between different cell types in human skin was analyzed using high‐throughput single‐cell expression profiling data (GSE150672). The results were visualized using the ggplot2 package.

### 4.18 Statistical Analysis

Each experiment was performed with three or more independent biological replicates. All data were tested for normal distribution before statistical analysis. For comparisons involving only two groups, statistical significance was determined using a two‑tailed unpaired Student's t‑test. For comparisons involving three or more groups, a one‑way analysis of variance (ANOVA) was performed, followed by Tukey's post‑hoc test for multiple pairwise comparisons. In all cases, a *p*‑value < 0.05 was considered statistically significant. Specific p‑values and F‑values are indicated in the figures or their respective legends (e.g., *p < 0.05, **p < 0.01, *p < 0.001). Graphs were generated using GraphPad Prism 10.1.2 (GraphPad Software, USA).

## Author Contributions

**Yaqing Wen**: conceptualization, methodology, Writing – original draft, formal analysis, visualization, investigation. **Jianjian Zhu**: investigation. **Jiangfeng Huang**: software. **Li Lei**: supervision. **Lan Zhang**: investigation, methodology. **Shu Zhou**: data curation. **Zhen Tang**: investigation. **Chuhan Fu**: project administration. **Qinghai Zeng**: funding acquisition, writing – review and editing, conceptualization. **Yuanyuan Wan**: conceptualization, methodology, visualization, writing – original draft, formal analysis, investigation. **Keyi Zhang**: validation. **Ling Jiang**: project administration. **Jing Chen**: writing – review and editing, resources. **Yushan Zhang**: methodology, formal analysis, visualization, conceptualization, writing – review and editing, investigation.

## Funding

High level Health Talents in Hunan Province (20240304082); Health Research Project of Hunan Provincial Health Commission (20256824); Natural Science Foundation of Hunan Province (2024JJ7010); Fundamental Research Funds for the Central Universities of the Central South University (No. 2026ZZTS0102).

## Conflicts of Interest

The authors declare no conflicts of interest.

## Supporting information




**Supporting File**: advs76820‐sup‐0001‐SuppMat.docx.

## Data Availability

The data that support the findings of this study are available from the corresponding author upon reasonable request.
