# Supplementary material for: Heat Stress Promotes Fibroblast‐Derived WNT5A Secretion Through m6A Modification to Activate Melanogenesis
Source: Adv Sci (Weinh). 2026 Jul 27:e76820. Online ahead of print. doi: 10.1002/advs.76820 (PMC13403720; doi:10.1002/advs.76820)
Supplement: Supplementary file 1 — Supporting File: advs76820‐sup‐0001‐SuppMat.docx. [file ADVS-9999-e76820-s001.docx]

**This file includes:**

Materials and methods

Figure S1 to S9

Tables S1 to S6

MATERIALS AND METHODS

**Western blot**

The total cellular protein was extracted using RIPA Lysis Buffer (Biosharp, China, #BL1321A) supplemented with 10% protease inhibitor (Roche, Switzerland, #04693159001) and 10% phosphatase inhibitor cocktail (Roche, Switzerland, #04906837001) and quantified using a BCA protein assay kit (KeyGEN Biotec, China, #KGPBCA). After blocking with 5% milk solution (BioFroxx，Germany, #1172GR100), the blots were incubated overnight with primary antibodies against TYR (ZENBIO, China, #121392)，TYRP1 (ZENBIO, China, #382326), DCT (ZENBIO, China, #821374), MITF (ZENBIO, China, #R24980), PMEL17 (Santa cruz, USA, #sc-393094), WANT5A (Proteintech, China, #55184-1-AP), FZD10 (Proteintech, China, #18175-1-AP), β-catenin (Proteintech, China, #51067-2-AP),p-β-catenin(Proteintech, China,# 80067-1-RR), METTL3 (Proteintech, China, #15073-1-AP), YTHDC1 (Proteintech, China, #29441-1-AP), or GAPDH (Cell Signaling Technology, USA, #5174S) at 4 ℃. All antibodies were diluted to1:1000, and the anti-GAPDH antibody was diluted to1:2000 with western primary antibody dilution buffer (#BL1027A, Biosharp, China). The membranes were washed with TBS-T buffer and then incubated for one hour with the appropriate secondary antibodies. For total protein detection, a goat anti-rabbit (1:10000; Zenbio, China, #511203) or goat anti-mouse (1:10000; Zenbio, China, #511103) secondary antibody was used. The positive bands were detected by enhanced chemiluminescence (ECL) using the ECL kit (Biosharp, China, #BL523A), per the manufacturer’s instructions.

**Co-immunoprecipitation**

Co-immunoprecipitation (Co-IP) was performed using an antibody cross‑linking immunoprecipitation kit (Beyotime, China, #P2180S). Briefly, for exogenous Co‑IP, cells transfected with FLAG‑FZD10 (Genma,China) were lysed and incubated with Flag antibody (Affinit, China, #T0053). For endogenous Co‑IP, human skin explants were lysed and incubated with WNT5A antibody (Santa Cruz, #SC‑365370). Protein A/G‑agarose beads were added for 2 h at room temperature. Beads were then washed, and bound proteins were eluted and analyzed by Western blotting. For detection, anti‑mouse IgG (AlpSdAbs®, #001‑100‑005) and anti‑rabbit IgG (AlpSdAbs®, #025‑100‑005) were used as secondary antibodies.

**Quantitative reverse transcription-polymerase chain reaction (RT-qPCR)**

The total cellular RNA was extracted using Fast total RNA extraction kit (Fastagen, China, #220011), and reverse-transcribed into cDNA using a reverse transcription kit (Vazyme, China, #R223-01), as per manufacturer’s instructions. The reverse transcription product was amplified by real-time PCR and the data were analyzed by the 2-ΔΔCt method. The primers used for amplification are shown in Supplementary **Table S4**.

**Cell Counting Kit-8 (CCK-8)**

Cell viability was evaluated using the CCK8 assay. Briefly, the cells were plated in 96-well plates at the density of 2000-3000 cells/well, and treated with different temperatures (37-43 ℃) for 1 hour daily for 3 consecutive days or varying STM2457 concentrations for 4 consecutive days. Ten microliters CCK8 (Biosharp, China, #BS350B) solution was added to each well and the cells were incubated for 2 h. The absorbance at 450 nm was measured using a microplate reader (PerkinElmer EnVision xcite, UK).

**Plasmid transfection**

The METTL3 overexpression plasmid (transcript NM_019852.5) was constructed in the pcDNA3.1(+) vector by Genma (China), with an empty vector as negative control. FB or MNT1 cells were seeded in plates. At 50–60% confluence, cells were washed twice with PBS and overlaid with Opti‑MEM. Lipofectamine 3000 and P3000 reagent were diluted separately in Opti‑MEM, mixed, and incubated at room temperature. The transfection mixture was then added to the cells and gently mixed. Cells were cultured under standard conditions for subsequent assays.

**Tyrosinase Fluorescent Probe**

Tyrosinase fluorescent probe can be used to detect tyrosinase activity in living cells. When the cell confluence in the well plate reached 40-50%, the culture medium was discarded and the cells were washed thrice with PBS buffer. Adding tyrosinase fluorescent probe to each well, and put in 37 ℃ incubator for 30 minutes under light protection. After incubation, discard the tyrosinase fluorescent probe solution, washed twice with PBS, and the cells were covered with PBS buffer pre-warmed to 37 ℃. After UV excitation, the tyrosinase activity in the living cells was observed by inverted fluorescence microscope.

**Immunofluorescence**

When the cell confluence in the well plate reached 40-50%, the culture medium was discarded and the cells were washed thrice with PBS buffer. Adherent cells were fixed with 4% paraformaldehyde (Servicebio, China, #G1101-500ML), permeabilized with 0.5% Triton X-100 liquid (G-clone, China, #CS9013). After blocking with 1% BSA (BioFroxx, Germany, #4240GR005), the blots were incubated overnight with primary antibodies against PMEL17 (Santa cruz, USA, #sc-393094), WNT5A (Proteintech, China, #55184-1-AP), FZD10 (Proteintech, China, #18175-1-AP), β-catenin (Proteintech, China, #51067-2-AP) or m6A (ABclonal, China, #A19841). Followed by fluorescent secondary antibodies (Affinity, Australia, #S0017, #S0018, #S0006). After counterstaining with DAPI (Biosharp, China, #BS097), the cells were observed under a confocal fluorescence microscope (LSM800; Zeiss, Oberkochin, Germany).

**Multiplex immunohistochemistry (mIHC)**

Staining was performed according to the Multiplex Fluorescence Staining Kit (Aifang biological, China, #AFIHC024). Sections were baked at 65 °C, deparaffinised in three changes of dewaxing agent, and rehydrated through graded ethanol to distilled water. Antigen retrieval was performed by microwave heating in retrieval buffer with alternating medium and medium‑low power cycles. After cooling to room temperature, slides were washed with PBS. Endogenous peroxidase activity was quenched with 3 % H_2_O_2_ in the dark, followed by PBS washes. Tissue sections were circled with a hydrophobic pen, blocked with 3 % BSA at room temperature, and incubated with the first primary antibody at 4 °C. After washing, a polymer‑HRP secondary antibody (ready‑to‑use, host‑matched) was applied in the dark, followed by incubation with the corresponding tyramide‑based fluorophore at room temperature and PBS washes. The antibody complex was then stripped by immersing the slides in retrieval buffer at 100 °C. The blocking, second primary antibody, polymer‑HRP, and a second spectrally distinct tyramide fluorophore were applied following the same sequence. Nuclei were counterstained with DAPI at room temperature in the dark. After final PBS washes, slides were mounted with an anti‑fade mounting medium and imaged using a fluorescence microscope.


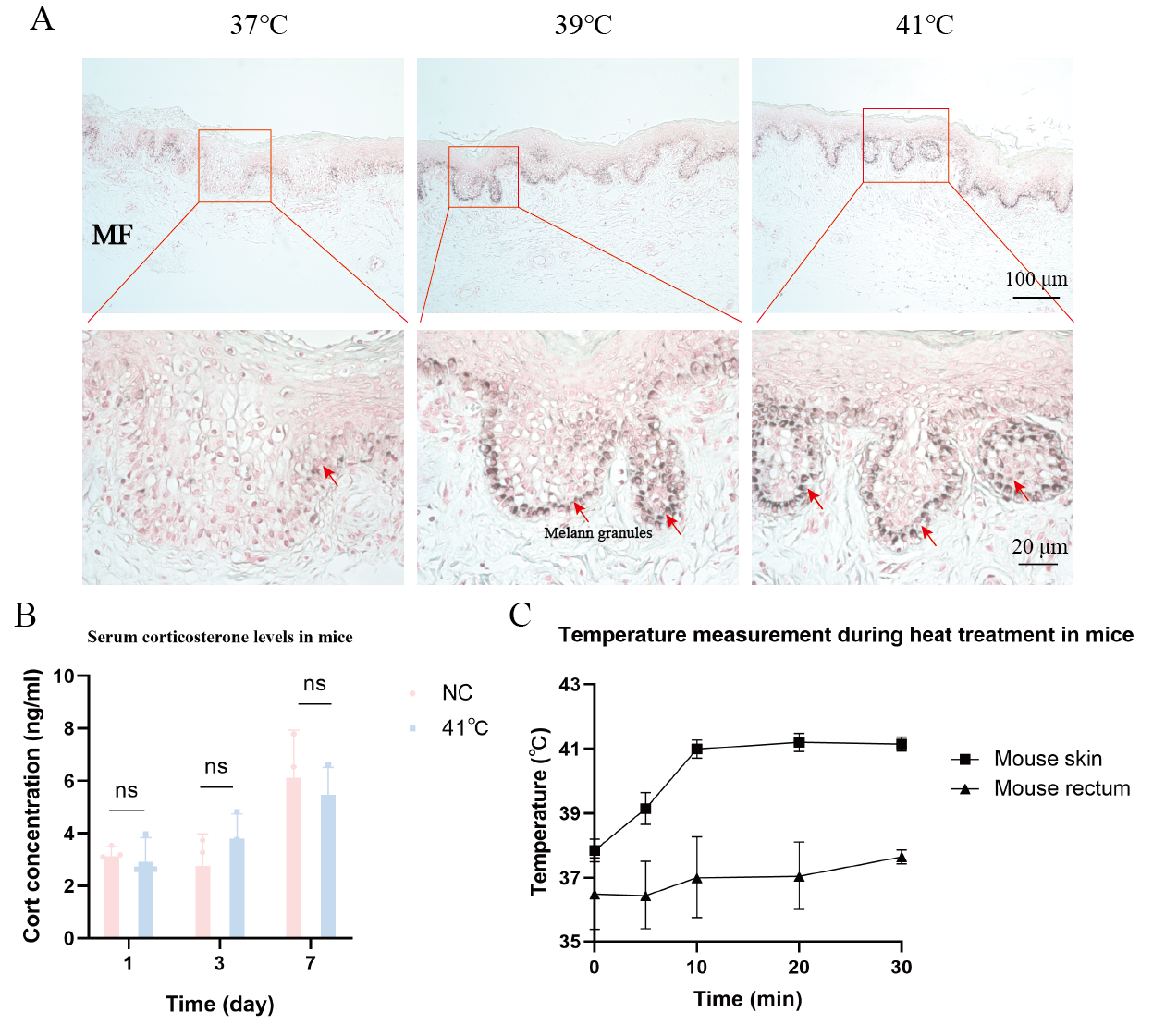


Figure S1.

**Heat stress promotes melanogenesis.** (A) Masson-Fontana staining was performed to assess melanin content in cultured human skin tissues after heat treatment (Scale bar = 100 μm, 20 μm). (B) Serum corticosterone concentrations in mice were quantified using an ELISA kit. Statistical significance was analyzed using Student’s t-test. (C) During heat exposure in mice, skin and rectal temperatures of the mice were recorded at 0, 10, 20, and 30min post heat treatment.


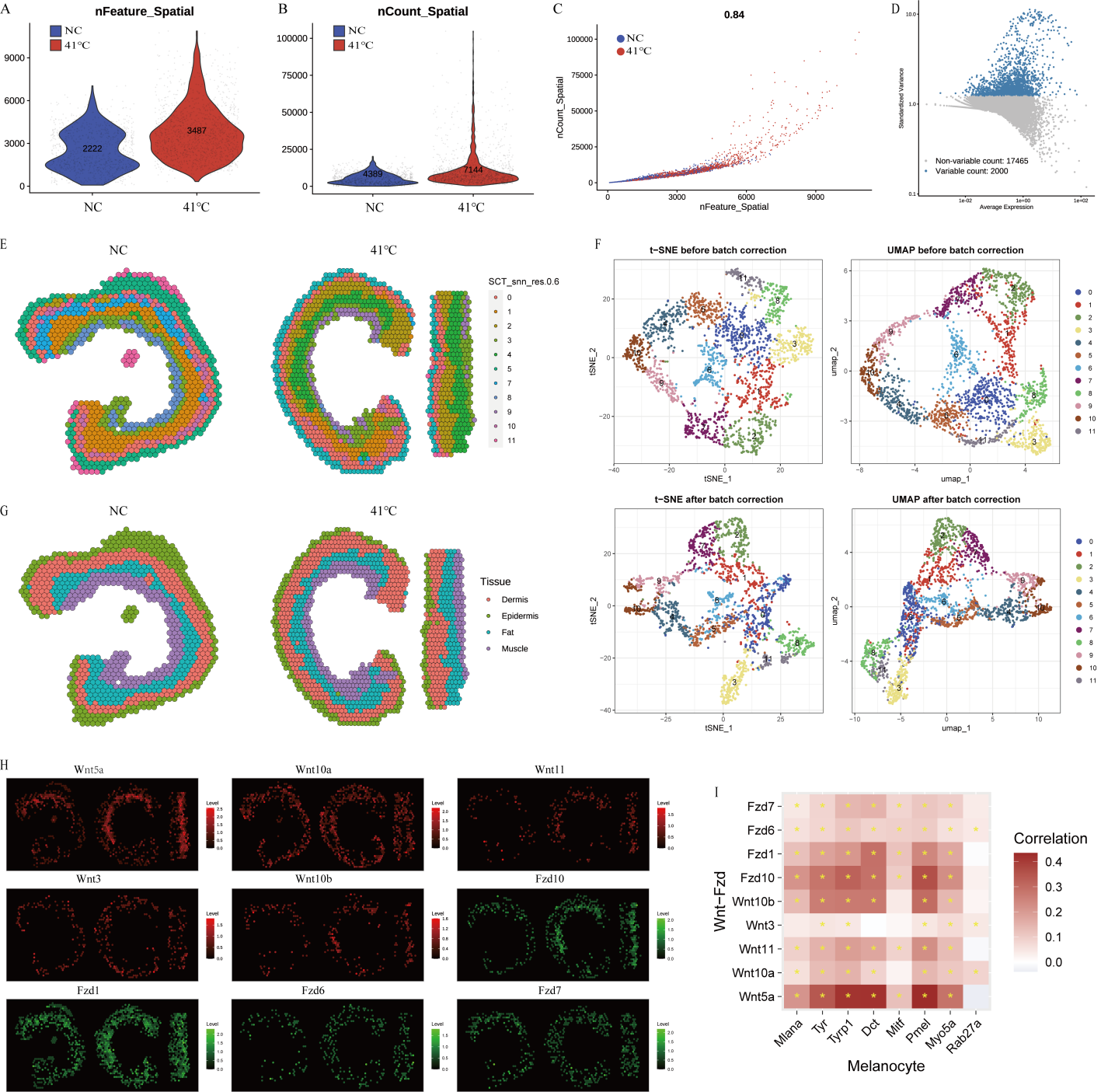


Figure S2.

**Supplementary quality control and downstream analysis of spatial transcriptomic data, related to Figures 2 and 3.** (A) Violin plots showing the distribution of the number of detected genes per spot (nFeature_Spatial) in the NC and 41 ℃ groups, with median values indicated. (B) Violin plots showing the distribution of UMI counts per spot (nCount_Spatial) in the NC and 41 ℃ groups, with median values indicated. (C) Quality-control scatter plot depicting the correlation between detected gene numbers (nFeature_Spatial) and UMI counts (nCount_Spatial) across spatial spots. (D) Scatter plot illustrating the number and distribution of variable genes. (E) Clustering of spatial transcriptomic data at a resolution parameter of 0.6. (F) Comparison of t-SNE and UMAP embeddings colored by spot clusters before and after batch correction, demonstrating improved integration and reduced batch effects. (G) Tissue types were classified into epidermis, dermis, fat, and muscle and displayed as a spatial scatter plot. (H) The spatial localization and expression of Wnt ligands and Fzd receptors that show statistically significant differences between the NC group and the 41 ℃ group. (I) Correlation between Wnt ligands, Fzd receptors, and melanogenesis-associated regulatory genes (Mlana, Tyr, Tyrp1, Dct, Mitf, Pmel, and Myo5a) (Pearson correlation analysis, *P < 0.05).


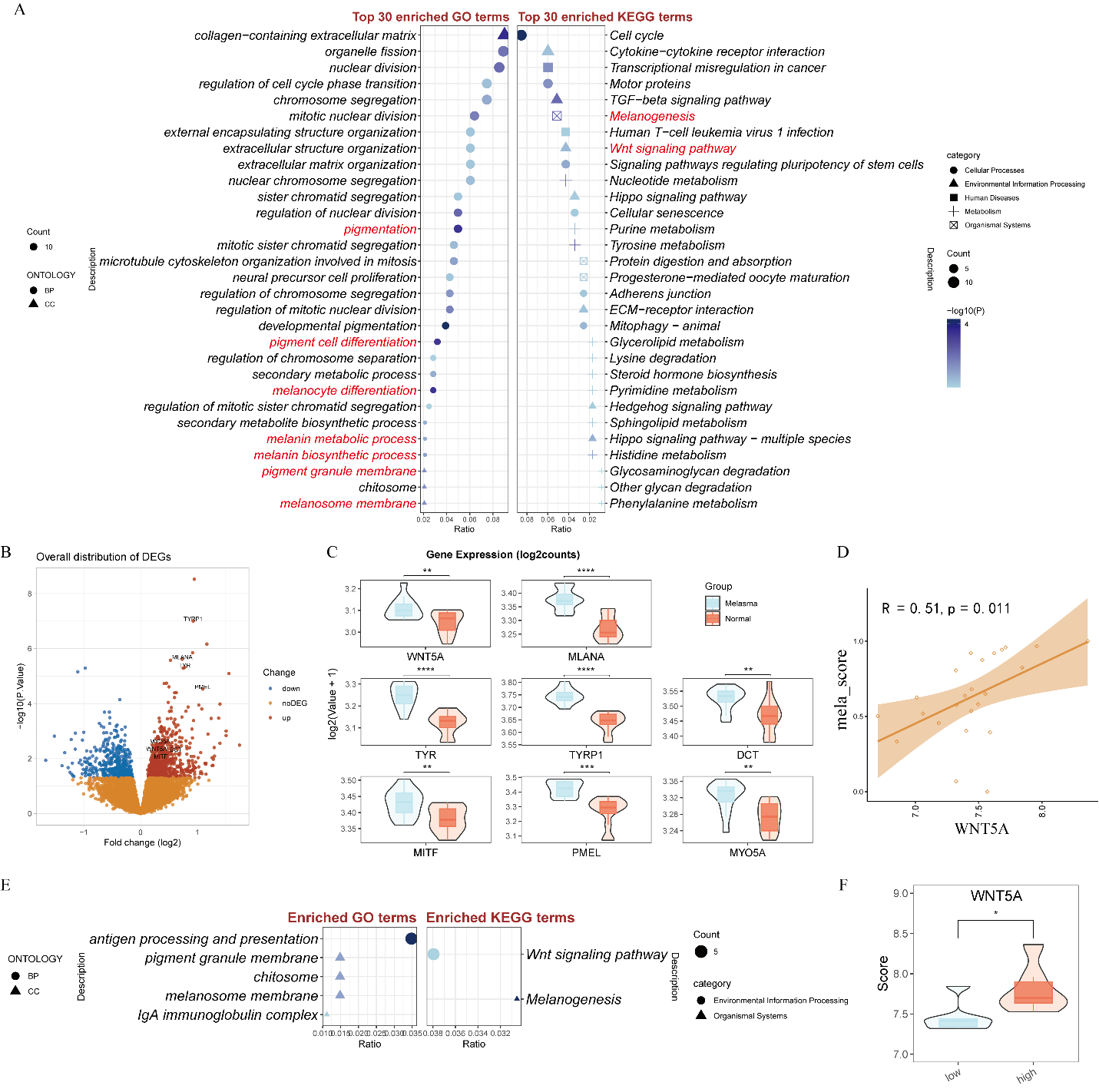


Figure S3.

**Melasma RNA-seq transcriptome analysis.** (A) Top 30 pathways of GO, KEGG enrichment results. (B) Differential expression volcano plots; (C) Violin plots demonstrating the expression values of WNT5A and melanogenesis-associated regulatory genes between the two groups (*P < 0.05; **P < 0.01; ***P < 0.001). (D) Correlation between WNT5A and melanogenesis-associated regulatory gene sets. (E) GO, KEGG enrichment pathways. (F) Violin plots demonstrating the expression values of the WNT5A genes in the TYR low expression and high expression groups (*P < 0.05).


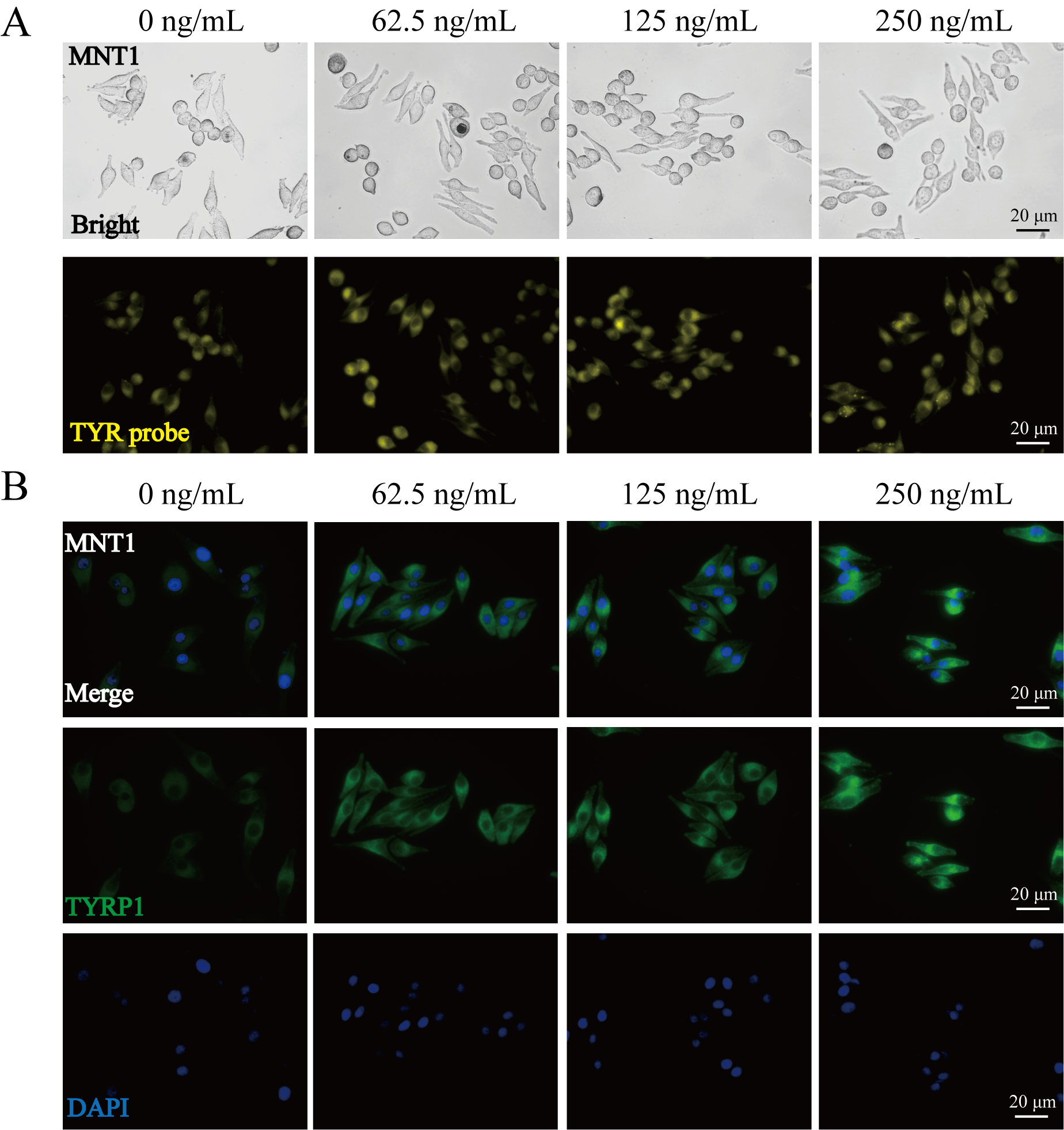


Figure S4.

**WNT5A promotes melanogenesis in melanocytes.** (A)TYR-probe detecting TYR activity in MNT1 cells after 2 days of WNT5A treatment (scale bar = 20 μm). (B) Immunofluorescence showing TYRP1 expression in MNT1 cells after 2 days of WNT5A treatment (scale bar = 20 μm).


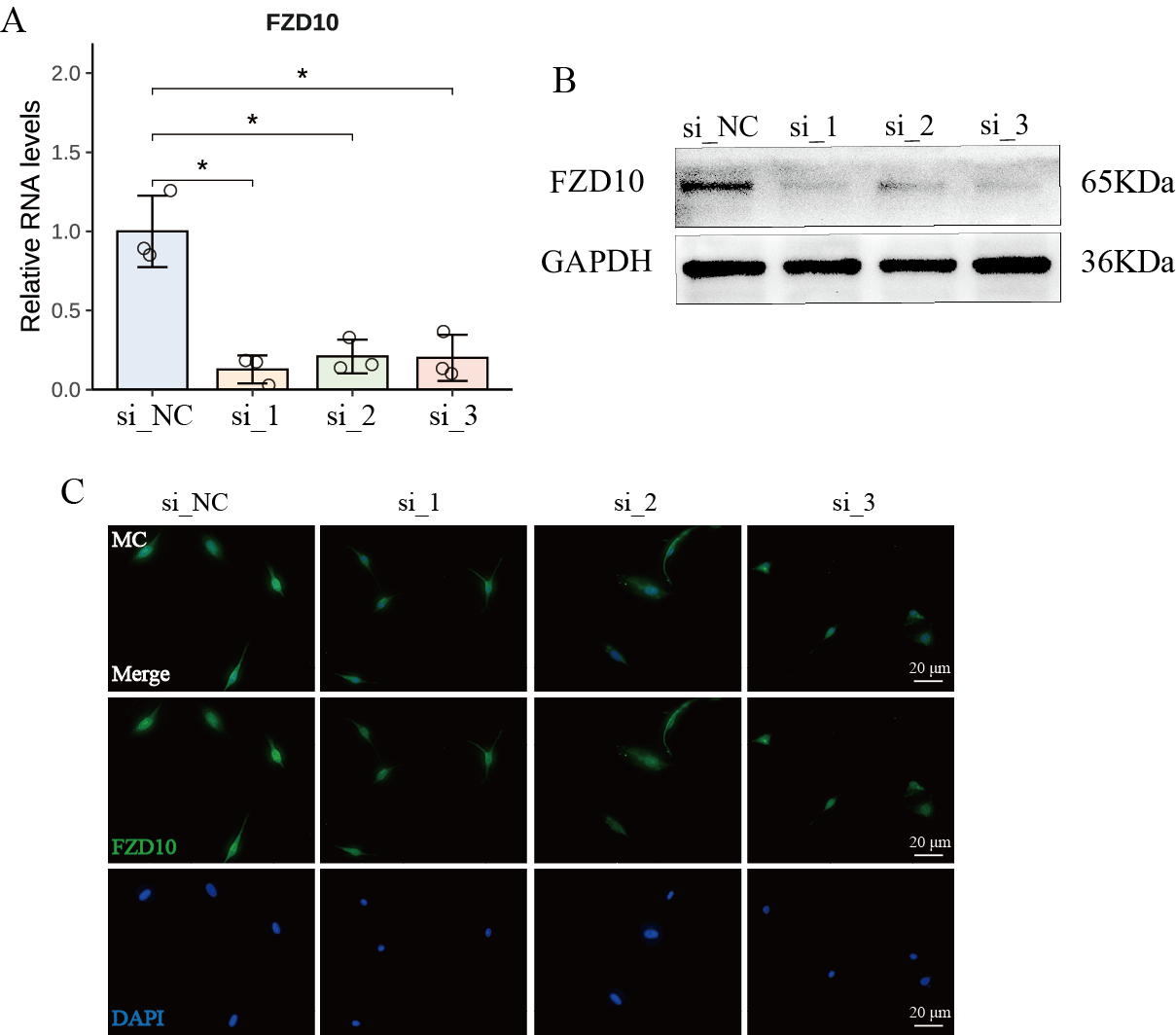


Figure S5.

**Validation of FZD10 siRNA knockdown efficiency.** (A) RT-qPCR analysis of FZD10 mRNA expression in MNT1 cells following FZD10 knockdown (si_1、si_2、si_3) (*P < 0.05). (B) Western blot analysis of FZD10 protein expression in MNT1 cells following FZD10 knockdown (si_1、si_2、si_3). (C) Immunofluorescence showing FZD10 protein expression in MC cells following FZD10 knockdown (si_1、si_2、si_3) (scale bar = 20 μm).


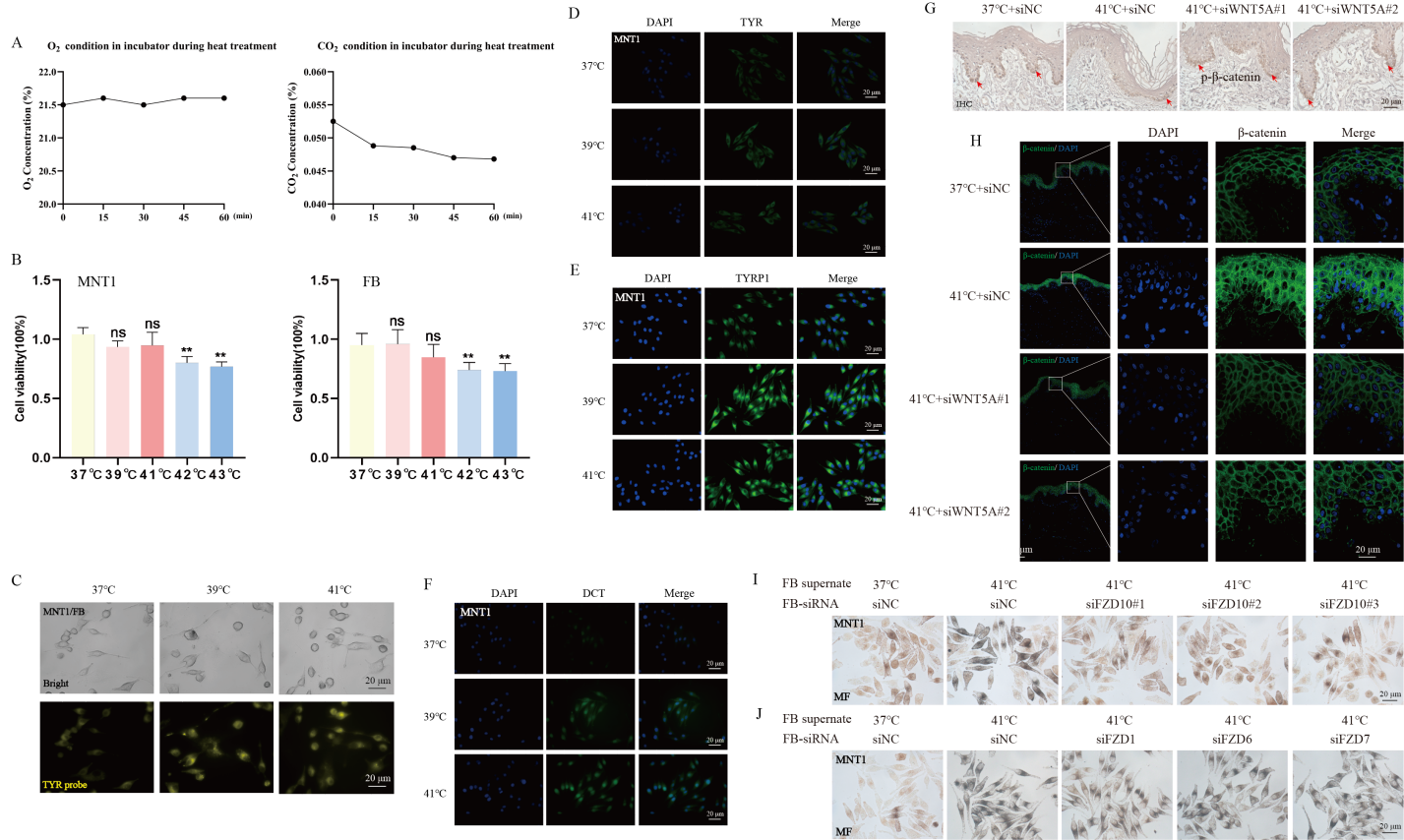


Figure S6.

**Heat-stressed fibroblast supernatant promotes melanocyte pigmentation.** (A) O2 and CO2 concentrations were measured at 0, 15, 30, 45, and 60 min in the constant-temperature incubator during cell heat treatment. (B) Cell viability of MNT1 and FB cells after treatment at different temperatures, assessed by CCK-8 assay. One-way ANOVA with Tukey's post-hoc test revealed significant differences among temperature groups for both cell lines (MNT1: F (4, 10) = 8.286; FB: F (4, 10) = 6.840). **P < 0.01. (C) TYR-probe detecting TYR activity in MNT1 cells within the co-culture system after heat stress (Scale bar = 20 μm). (D) Immunofluorescence localization to observe the fluorescence intensity of TYR in MNT1 cells after FB supernatant treatment (Scale bar = 20 μm). (E) Immunofluorescence localization to observe the fluorescence intensity of TYRP1 in MNT1 cells after FB supernatant treatment (Scale bar = 20 μm). (F) Immunofluorescence localization to observe the fluorescence intensity of DCT in MNT1 cells after FB supernatant treatment (Scale bar = 20 μm). (G) Immunohistochemistry images showing p-β-catenin protein expression in human skin sections following 5 days of heat stress with WNT5A knockdown (Scale bar = 20 μm). (H) Immunofluorescence analysis of β-catenin protein expression (green) in human skin sections after 5 days of heat stress with WNT5A-knockdown (scale bar = 100 μm, 26 μm). (I) Masson-Fontana staining showing melanin content in MNT1 cells after 2 days of combined treatment with FB supernatant and FZD10 knockdown (scale bar = 20 μm). (J) Masson-Fontana staining showing melanin content in MNT1 cells after 2 days of combined treatment with FB supernatant and FZD1/6/7 knockdown (scale bar = 20 μm).


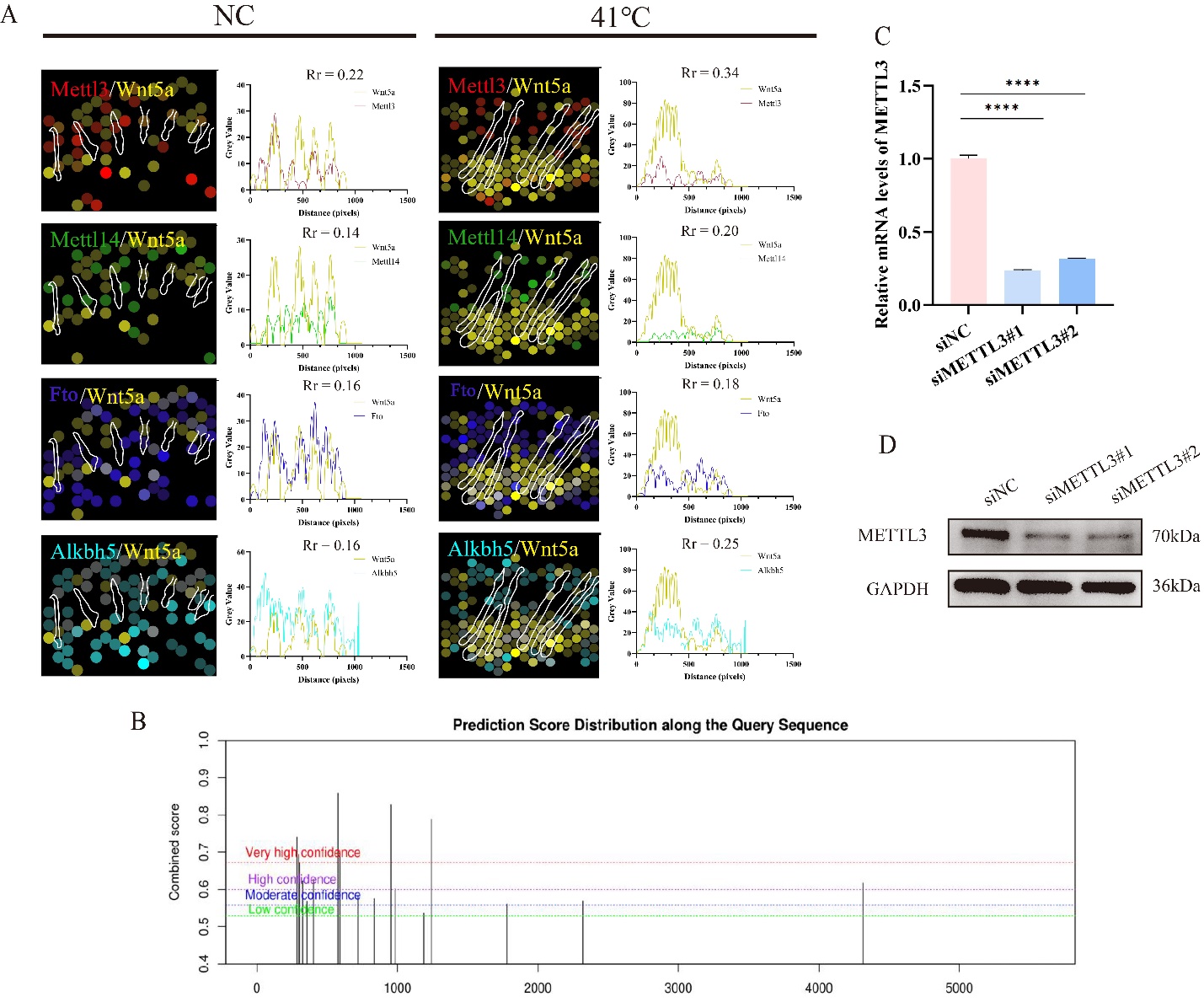


Figure S7.

**Functional validation of METTL3-m6A-WNT5A axis in heat-induced melanogenesis, related to Figure 7.** (A) Colored dot visualization of Wnt5a, and m6A writers and erasers genes (Mettl3, Mettl14, Fto, Alkbh5) in hair follicle regions of NC and 41 ℃ groups. Notably, Wtap, another core component of the m6A methyltransferase complex, was not detected among the spatially analyzed genes in this study. (B) Prediction of m6A modification sites on WNT5A mRNA using SRAMP (http://www.cuilab.cn/sramp/). (C) RT-qPCR analysis of METTL3 mRNA expression in FB cells following METTL3 knockdown. One-way ANOVA followed by Tukey’s post-hoc test (F (2, 6) = 2587, ****p < 0.0001). (D) Western blot analysis of METTL3 protein expression in METTL3-knockdown FB cells following heat stress.


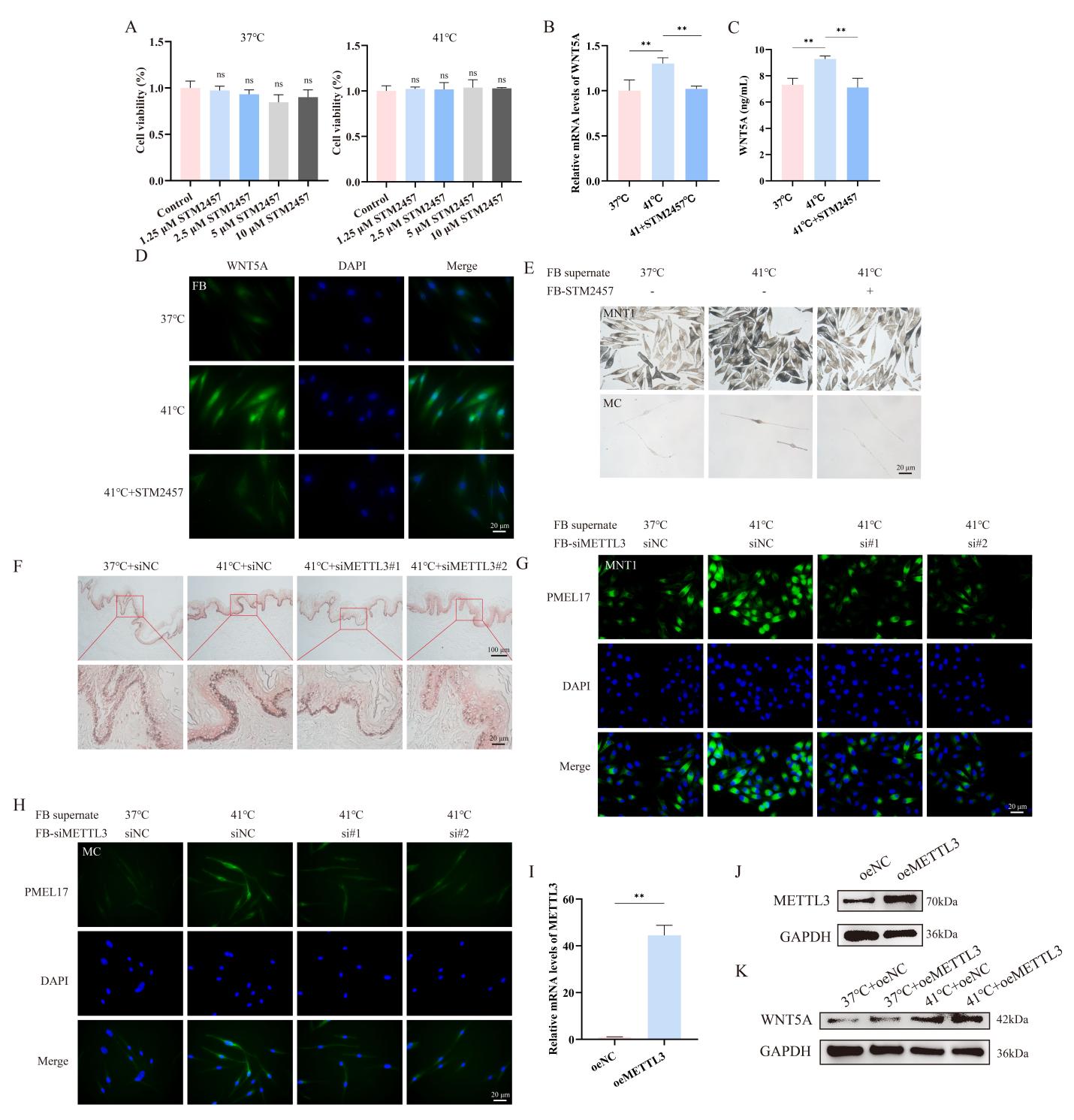


Figure S8.

**Functional validation of METTL3-m6A-WNT5A axis in heat-induced melanogenesis, related to Figure 8.** (A) CCK8 of FB cells treated with indicated concentrations of STM2457 for 96h at 37 ℃ and 41 ℃. One-way ANOVA followed by Tukey’s post-hoc test, no significant differences were found. (B) RT-qPCR analysis of WNT5A mRNA expression in FB cells pretreated with 2.5 μM STM2457 followed by heat stress. One-way ANOVA followed by Tukey’s post-hoc test (F (2, 6) = 13.43, **p < 0.01). (C) ELISA quantification of WNT5A levels in conditioned supernate from heat-stressed FB cells pretreated with 2.5 μM STM2457. One-way ANOVA followed by Tukey’s post-hoc test (F (2, 6) = 16.53, **p < 0.01). (D) Immunofluorescence analysis of WNT5A in FB cells pretreated with 2.5 μM STM2457 followed by heat stress (scale bar = 20 μm). (E) Masson-Fontana melanin staining of MNT1 and MC cells treated for 2 days with conditioned supernate from heat-stressed FB cells pretreated with 2.5μM STM2457 (scale bar = 20 μm). (F) Masson-Fontana staining showing melanin content in human skin sections after 5 days of heat stress with METTL3 knockdown (scale bar = 100 μm, 20 μm). (G) Immunofluorescence analysis of PMEL17 expression in MNT1 cells treated for 2 days with conditioned supernate from METTL3-knockdown FB cells following heat stress (scale bar = 20 μm). (H) Immunofluorescence analysis of PMEL17 expression in MC cells treated for 2 days with conditioned supernate from METTL3-knockdown FB cells following heat stress (scale bar = 20 μm). (I) RT-qPCR analysis of METTL3 mRNA expression in FB cells following METTL3-overexpressing. Statistical significance was analyzed using Student’s t-test (**P < 0.01). (J) Western blot analysis of METTL3 protein expression in METTL3-overexpressing FB cells following heat stress. (K) Western blot analysis of WNT5A protein expression in FB cells following METTL3-overexpressing.


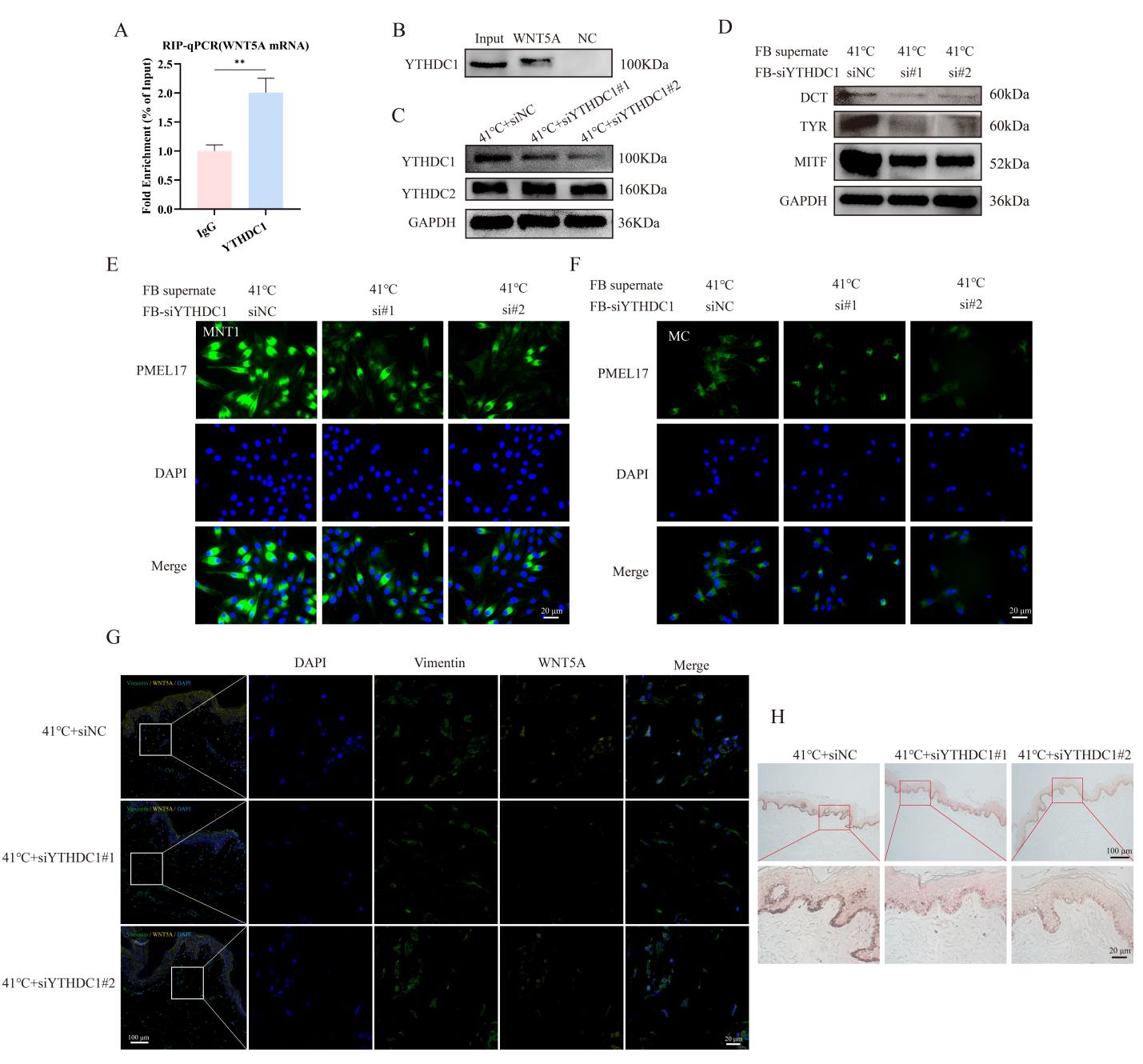
Figure S9.

**Functional validation of METTL3/YTHDC1-m6A-WNT5A axis in heat-induced melanogenesis, related to Figure 9.** (A) RIP-qPCR analysis of the interaction between YTHDC1 protein and WNT5A mRNA. Statistical significance was analyzed using Student’s t-test (**P < 0.01). (B) RNA pull-down analysis of the interaction between YTHDC1 protein and WNT5A mRNA. (C) Western blot analysis of YTHDC1/YTHDC2 protein expression in YTHDC1-knockdown FB cells following heat stress. (D) Western blot analysis of DCT, TYR and MITF protein expression in MNT1 cells treated for 2 days with conditioned supernate from YTHDC1-knockdown FB cells following heat stress. (E) Immunofluorescence analysis of PMEL17 expression in MNT1 cells treated for 2 days with conditioned supernate from YTHDC1-knockdown FB cells following heat stress (scale bar = 20 μm). (F) Immunofluorescence analysis of PMEL17 expression in MC cells treated for 2 days with conditioned supernate from YTHDC1-knockdown FB cells following heat stress (scale bar = 20 μm). (G) Immunofluorescence analysis of WNT5A protein expression (yellow) in vimentin-positive fibroblasts (green) in human skin sections after 5 days of heat stress with YTHDC1-knockdown (scale bar = 100 μm, 20 μm). (H) Masson-Fontana staining showing melanin content in human skin sections after 5 days of heat stress with YTHDC1-knockdown (scale bar = 100 μm, 20 μm).

Table S1.

Clinical characteristic of patients.

| Patient No. | Age | Sex | Skin Type | Duration of vitiligo (months) | Type of vitiligo | Stage of vitiligo | Location of lesions | Family of history | Previous Treatment | Conventional therapy during follow-up | SCS treatment during follow-up | |
| --- | --- | --- | --- | --- | --- | --- | --- | --- | --- | --- | --- | --- |
|  |  |  |  |  |  |  |  |  |  |  | Type & Route | Dose & Duration |
| 1 | 43 | F | III | 1 | NS | Progressive | Trunk | Yes | No | SCS+TCS+TCI+CKT+308 laser | Methylprednisolone (Oral) | Week 1-4: 12 mg/d; Week 5-8: 8 mg/d; Week 9-12: 4 mg/d |
| 2 | 30 | M | IV | 6 | S | Progressive | Trunk | No | Yes | SCS+OQT +TCS+CKT +NB-UVB | Compound Betamethasone Injection (Intramuscular) | 1 mL, single dose in week 1 |
| 3 | 10 | M | IV | 2 | S | Progressive | Upper extremity | No | Yes | TCI+CKT+308 laser | / | / |
| 4 | 22 | F | IV | 12 | S | Stable | Face, neck | No | Yes | TCS+TCI+CKT+308 laser | / | / |
| 5 | 4 | F | IV | 24 | NS | Progressive | Trunk | No | No | SCS+TCS+CKT+308 laser+CO_2_ laser | Methylprednisolone (Oral) | 8 mg/d for 20 days, 4 mg/d for 20 days, then stopped |
| 6 | 15 | F | IV | 36 | NS | Progressive | Trunk | No | No | SCS+TCI+CKT+308 laser+CO_2_ laser | Betamethasone Injection (Intramuscular) | 1 mL, single dose in week 1 |

M, Male; F, female; NS, non-segmental; S, segmental; SCS, systemic corticosteroids; OQT, oral qubaibabuqi tablet; TCS, topical corticosteroids; TCI, topical calcineurin inhibitors; CKT, compound kaliziran tincture; 308 laser, 308 nm excimer laser; NB-UVB, narrow-band ultraviolet B; CO_2_ laser, fractional CO_2_ laser.

Table S2

Genes encoding upstream ligands and receptors involved in melanogenesis-related pathways.

| Ligands | Receptors | Ligands | Receptors | Ligands | Receptors | Ligands | Receptors | Ligands | Receptors | Ligands | Receptors | Ligands | Receptors | Ligands | Receptors | Ligands | Receptors | Ligands | Receptors | Ligands | Receptors |
| --- | --- | --- | --- | --- | --- | --- | --- | --- | --- | --- | --- | --- | --- | --- | --- | --- | --- | --- | --- | --- | --- |
| Wnt1 | Fzd1 | Fgf1 | Fgfr1 | Bmp1 | Bmper | Tgfa | Tgfbr1 | Notch1 | Dll1 | Nrg1 | Erbb2 | Edn1 | Ednra | Scf | Kit | Hgf | Met | Pomc | Mc1r | Ngf | Ngfr |
| Wnt10a | Fzd2 | Fgf10 | Fgfr2 | Bmp10 | Bmpr1a | Tgfb1 | Tgfbr3 | Notch2 | Dll3 | Nrg2 | Erbb3 | Edn2 | Ednrb |  |  |  |  |  | Mc4r |  |  |
| Wnt10b | Fzd3 | Fgf11 | Fgfr3 | Bmp15 | Bmpr1b | Tfrb1i1 | Tgfbr3l | Notch3 | Dll4 | Nrg3 |  | Edn3 |  |  |  |  |  |  |  |  |  |
| Wnt11 | Fzd4 | Fgf12 | Fgfr4 | Bmp2 | Bmpr2 | Tgfb2 | Tgfbrap1 | Notch4 | Jag1 | Nrg4 |  |  |  |  |  |  |  |  |  |  |  |
| Wnt16 | Fzd5 | Fgf13 | Fgfrl1 | Bmp2k |  | Tgfb3 |  |  | Jag2 |  |  |  |  |  |  |  |  |  |  |  |  |
| Wnt2 | Fzd6 | Fgf14 |  | Bmp3 |  | Tgfbi |  |  |  |  |  |  |  |  |  |  |  |  |  |  |  |
| Wnt2b | Fzd7 | Fgf15 |  | Bmp4 |  |  |  |  |  |  |  |  |  |  |  |  |  |  |  |  |  |
| Wnt3 | Fzd8 | Fgf16 |  | Bmp5 |  |  |  |  |  |  |  |  |  |  |  |  |  |  |  |  |  |
| Wnt3a | Fzd9 | Fgf17 |  | Bmp6 |  |  |  |  |  |  |  |  |  |  |  |  |  |  |  |  |  |
| Wnt4 | Fzd10 | Fgf18 |  | Bmp7 |  |  |  |  |  |  |  |  |  |  |  |  |  |  |  |  |  |
| Wnt5a |  | Fgf2 |  | Bmp8a |  |  |  |  |  |  |  |  |  |  |  |  |  |  |  |  |  |
| Wnt5b |  | Fgf20 |  | Bmp8b |  |  |  |  |  |  |  |  |  |  |  |  |  |  |  |  |  |
| Wnt6 |  | Fgf21 |  |  |  |  |  |  |  |  |  |  |  |  |  |  |  |  |  |  |  |
| Wnt7a |  | Fgf22 |  |  |  |  |  |  |  |  |  |  |  |  |  |  |  |  |  |  |  |
| Wnt7b |  | Fgf23 |  |  |  |  |  |  |  |  |  |  |  |  |  |  |  |  |  |  |  |
| Wnt8a |  | Fgf3 |  |  |  |  |  |  |  |  |  |  |  |  |  |  |  |  |  |  |  |
| Wnt8b |  | Fgf4 |  |  |  |  |  |  |  |  |  |  |  |  |  |  |  |  |  |  |  |
| Wnt9a |  | Fgf5 |  |  |  |  |  |  |  |  |  |  |  |  |  |  |  |  |  |  |  |
| Wnt9b |  | Fgf6 |  |  |  |  |  |  |  |  |  |  |  |  |  |  |  |  |  |  |  |
|  |  | Fgf7 |  |  |  |  |  |  |  |  |  |  |  |  |  |  |  |  |  |  |  |
|  |  | Fgf8 |  |  |  |  |  |  |  |  |  |  |  |  |  |  |  |  |  |  |  |
|  |  | Fgf9 |  |  |  |  |  |  |  |  |  |  |  |  |  |  |  |  |  |  |  |
|  |  | Fgfbp1 |  |  |  |  |  |  |  |  |  |  |  |  |  |  |  |  |  |  |  |
|  |  | Fgfbp3 |  |  |  |  |  |  |  |  |  |  |  |  |  |  |  |  |  |  |  |

Table S3.

siRNA sequences.

| **Gene** | **sense（5'-3'）** | **antisense（5'-3'）** |
| --- | --- | --- |
| METTL3#1 | GCUCAACAUACCCGUACUATT | UAGUACGGGUAUGUUGAGCTT |
| METTL3#2 | CCUGCAAGUAUGUUCACUATT | UAGUGAACAUACUUGCAGGTT |
| FZD10#1 | CCCAUCCAGUUGCACGAGUUTT | AACUCGUGCAACUGGAUGGTT |
| FZD10#2 | GCUACAACAUGACUCGUAUTT | AUACGAGUCAUGUUGUAGCTT |
| FZD10#3 | CCAUCCUGAUCCUGGUCAUTT | AUGACCAGGAUCAGGAUGGTT |
| YTHDC1#1 | GAGUAACAACCAUGAGAAU | AUUCUCAUGGUUGUUACUC |
| YTHDC1#2 | GAAUUACCCUUCACUAAGU | ACUUAGUGAAGGGUAAUUC |
| WNT5A#1 | GUGGUCGCUAGGUAUGAAUTT | AUUCAUACCUAGCGACCACTT |
| WNT5A#2 | CGCGAAGACAGGCAUCAAATT | UUUGAUGCCUGUCUUCGCGTT |

Table S4.

PCR Primer sequences.

| **Gene** | **Forward primer sequences（5'-3'）** | **Reverse primer sequences（5'-3'）** |
| --- | --- | --- |
| GAPDH | CTCTGCTCCTCCTGTTCGAC | GCCCAATACGACCAAATCC |
| WNT5A | CTGGCAGGACTTTCTCAAGG | CTGGCAGGACTTTCTCAAGG |
| WNT5A (For MeRIP-qPCR) | AAGCCAATTCTTGGTGGTCG | TTCTTTGATGCCTGTCTTCGC |
| FZD10 | GCTCAAGTGCTCCCCGATTA | GCTCAAGTGCTCCCCGATTA |
| WTAP | ACTGGCCTAAGAGAGTCTGAAG | GTTGCTAGTCGCATTACAAGGA |
| ALKBH5 | GACAAGGAAGAGAACCGGCG | GCATCTTCACCTTTCGGGCA |
| METTL14 | AGGAACTGTGAAGCGTAGCA | AGCCAGCCTGGTCGAATTG |
| METTL3 | CTGCTTGGTTGGTGTCAAAGG | GCGAGTGCCAGGAGATAGTC |
| FTO | AATTCTATCAGCAGTGGCAGC | TGAGGATGCGAGATACCGGA |
| YTHDC1 | AACTGGTTTCTAAGCCACTGAGC | GGAGGCACTACTTGATAGACGA |
| YTHDF1 | CGTGGACACCCAGAGAACAAA | CCAATGGACGGCGGGTAATA |
| YTHDF2 | TGTTGGAGAAGCTTCGGTCC | ACCCGGCCATGTTTCAGATT |
| IGF2BP1 | CTTTGTAGGGCGTCTCATTGGC | CCTTCACAGTGATGGTCCTCTC |
| IGF2BP2 | GTTGGTGCCATCATCGGAAAGG | TGGATGGTGACAGGCTTCTCTG |
| IGF2BP3 | TCGTGACCAGACACCTGATGAG | GGTGCTGCTTTACCTGAGTCAG |

Table S5.

Information of GEO datasets.

| **GEO accession** | **Platform** | **Size** | **Source tissue** |
| --- | --- | --- | --- |
| GSE72140 | GPL570 | 48 | Human skin |
| GSE150672 | GPL18573 | 438 | Human skin |

Table S6

Template of informed consent form for thermotherapy in vitiligo treatment.

| **The Third Xiangya Hospital of Central South University**  **Informed Consent Form for Thermotherapy Treatment of Vitiligo** | | | |
| --- | --- | --- | --- |
| **Patient Name** | **Gender** | **Age** | **Medical Record No.** |
| **Disease Introduction and Treatment Recommendations**  The doctor has informed me that I have been diagnosed with vitiligo, and recommends thermotherapy treatment in addition to conventional therapy.  Disease Introduction: This condition is an autoimmune skin disorder with a complex etiology that has not been fully elucidated. It is generally believed that vitiligo occurs on a genetic background, with internal and external factors acting together to induce immune cells to destroy melanocytes, leading to the development of white patches. If left untreated, the patches may spread and worsen.  Thermotherapy: This therapy uses mild heat (around 41°C) applied to the skin lesions. It can promote local blood circulation and enhance drug absorption. Additionally, it activates the paracrine function of keratinocytes, promoting melanin production by melanocytes. It also inhibits the expression of key pathogenic factors in vitiligo, thereby achieving repigmentation of the affected areas.  Expected effect: Promotion of repigmentation of white patches. | | | |
| **Potential Risks and Countermeasures**  The doctor has informed me that thermotherapy may involve certain risks. Some uncommon risks may not be listed here. If I have specific questions, I may discuss them with my doctor.  1. I understand that any treatment involves risks.  2. I understand that any medication used may cause side effects, ranging from mild nausea, rash, etc., to severe allergic shock or even life-threatening events.  3. I understand the possible risks and limitations of this treatment, including:  Burning sensation, itching, local redness, blisters, erosions or ulcers, local infection, scar formation, hyperpigmentation, varying degrees of burns, and other uncommon or unforeseeable risks. Repigmentation of vitiligo requires repeated and regular treatment, and relapse or worsening remains possible.  **Specific Risks or Major Risk Factors**  I understand that based on my personal condition, I may experience the following specific complications or risks:  The doctor will take active measures if any of the above risks or unexpected events occur. | | | |
| **Patient’s Informed Consent**   - My doctor has informed me of the treatment method to be performed, the potential complications and risks associated with this treatment and its aftermath, alternative treatment options available, and has answered my questions regarding this treatment. - I agree that the doctor may adjust the planned treatment based on my condition during the course of therapy. - I understand that my treatment will be carried out by multiple healthcare professionals. - I agree that the research results (including images and clinical data) may be used for academic exchange and publication. - I have not been promised that the treatment will be 100% successful.   Patient’s signature Date：  If the patient is unable to sign, an authorized relative may sign here:  Authorized relative’s signature Relationship to patient：  Date：  **Physician’s Statement**  I have informed the patient of the treatment method to be performed, the potential complications and risks associated with this treatment and its aftermath, alternative treatment options available, and have answered the patient’s questions regarding this treatment.  Physician’s signature Date | | | |
